# Oxygen-responsive KoBdcA/KoBdcB/KoBpdA system regulates c-di-GMP levels to control bacterial cellulose biosynthesis and motility in *Kosakonia oryzendophytica* FY-07

**DOI:** 10.1128/spectrum.02292-25

**Published:** 2026-03-26

**Authors:** Xueqing Zhao, Yucheng Shi, Wenzhuo Tian, Yutong Tian, Chuxiao Hu, Ziye Guo, Ting Ma, Guoqiang Li, Ge Gao

**Affiliations:** 1 College of Life Sciences, Nankai University 117931, Tianjin, China; 2 Key Laboratory of Molecular Microbiology and Technology, Ministry of Education, Nankai University 616838 , Tianjin, China; 3 Tianjin Engineering Technology Center of Green Manufacturing Biobased Materials, Tianjin, China; 4 School of Life Science, Shanxi University 630971, Taiyuan, China; Rutgers The State University of New Jersey, Piscataway, New Jersey, USA

**Keywords:** c-di-GMP, diguanylate cyclases, phosphodiesterases, bacterial cellulose, bacterial motility

## Abstract

Cyclic di-GMP (c-di-GMP) acts as a bacterial second messenger that regulates various physiological processes, including biofilm formation, motility, and virulence. It is synthesized by diguanylate cyclases (DGCs) and degraded by phosphodiesterases (PDEs), with precise regulatory effects typically requiring the coordinated action of multiple DGCs and PDEs. *Kosakonia oryzendophytica* FY-07 (FY-07) exhibits high bacterial cellulose (BC) yield, rapid growth, and robustness and can produce BC under varying oxygen conditions; however, the underlying mechanism driving the phenotypic transition from cellulose-producing to motile states during fermentation has yet to be elucidated. In this study, we aimed to identify two DGCs, KoBdcA and KoBdcB, and one PDE, KoBpdA, whose encoding genes are located adjacent to the cellulose synthesis operon in FY-07. KoBdcA and KoBpdA form an antagonistic DGC-PDE pair that maintains intracellular c-di-GMP homeostasis. Deletion of either gene alters BC production and motility. In contrast, KoBdcB acts as an auxiliary component, and its deletion alone causes no phenotypic changes. KoBdcA regulates BC synthesis via an indirect interaction with BcsA mediated by KoBdcB. The Per-Arnt-Sim (PAS) domains of KoBdcA and KoBpdA bind hemin, enabling them to sense oxygen and modulate their enzymatic activities. Elevated DGC activity during early fermentation promotes BC production, while increased PDE activity later suppresses BC synthesis, shifting cells to a motile state.

**IMPORTANCE:**

In this study, we provide novel mechanistic insights into how synergistic interactions among multiple DGCs and PDEs, in response to oxygen sensing, control the dynamic switch between the sessile and motile states of biofilm-producing bacteria. The study findings provide theoretical insights into the localized regulation of c-di-GMP signaling in *K. oryzendophytica* and have significant implications for elucidating the metabolic regulatory network of c-di-GMP. These findings also provide valuable insights into the molecular interactions and mechanisms governing the association between *Kosakonia* species and their host plants.

## INTRODUCTION

Bacteria frequently encounter diverse environmental stresses during their life cycle, necessitating rapid sensing of external changes and behavioral adjustments to enhance adaptability (1). Cyclic di-GMP (c-di-GMP) is a universal bacterial second messenger regulating motility, adhesion, extracellular polysaccharide (EPS) production, cell cycle regulation, and virulence (2). Its intracellular level is regulated by the opposing activities of diguanylate cyclases (DGCs), which synthesize c-di-GMP via their Gly-Gly-Asp-Glu-Phe (GGDEF) domains, and phosphodiesterases (PDEs), which degrade it via Glu-Ala-Leu (EAL) or His-Asp-Gly-Tyr-Pro (HD-GYP) domains. DGC and PDE activities are commonly regulated by upstream sensory domains that enable responses to environmental signals. Common sensory modules include PAS domains, cGMP-specific phosphodiesterases/Adenylyl cyclases/FhlA (GAF) domains, membrane-spanning heme-binding domains of TonB-dependent transporters (MHYT), and hemerythrin domains, which detect oxygen, redox states, light, or other small-molecule ligands (3). These modules modulate intracellular c-di-GMP levels via ligand binding or conformational changes, thereby controlling phenotypic transitions (4, 5). Among the many environmental cues that influence bacterial physiology, oxygen is notable for its dual role as a metabolic substrate and signaling molecule. Its levels vary across environments, requiring bacteria to sense and adapt. Oxygen also regulates gene expression via heme- and redox-sensitive domains, controlling energy metabolism, motility, biofilm formation, and virulence (6, 7).

The complexity of the c-di-GMP network is further reflected in the large number of genes involved. For instance, the *Pseudomonas aeruginosa* PAO1 genome contains 42 relevant genes (8), *Escherichia coli* K-12 contains 29 (9), *Sinorhizobium meliloti* has 22 (10), and *Vibrio cholerae* contains up to 62 (11). Interactions among DGCs, PDEs, and their effectors form a signaling network that precisely regulates c-di-GMP, producing distinct physiological outcomes under diverse environmental conditions (12). For example, in *P. aeruginosa*, the PDE BifA interacts with PelD to locally modulate c-di-GMP and specifically regulate biofilm formation (13), whereas in *Pseudomonas fluorescens*, the DGC GcbC directly interacts with LapD to control cell surface attachment (14). These findings have led to the concept of “localized c-di-GMP signaling,” in which spatially restricted DGC-PDE modules precisely regulate specific cellular processes without altering global c-di-GMP levels (15). Such mechanisms underlie complex bacterial behaviors, including transitions between motile and sessile states.

c-di-GMP was first discovered in the obligate aerobic bacterium *Gluconacetobacter xylinus*, where it allosterically activates the cellulose synthase complex and promotes bacterial cellulose (BC) biosynthesis (16). *Kosakonia oryzendophytica* (*K. oryzendophytica*) FY-07 (formerly *Enterobacter* sp. FY-07) is a gram-negative, facultatively anaerobic bacterium that exhibits pronounced plant growth-promoting traits, including nitrogen fixation, phosphate solubilization, and phytohormone secretion. The discovery of FY-07 challenged the traditional notion that BC synthesis occurs exclusively under aerobic conditions (17). Remarkably, FY-07 is capable of producing BC under aerobic, microaerobic, and anaerobic environments, making it a promising candidate for large-scale BC production. Previous studies indicated large fluctuations in oxygen levels during BC fermentation by FY-07, suggesting that it may respond to oxygen signals (18). It has been reported that oxygen modulates intracellular c-di-GMP concentrations via DGC/PDE regulatory systems, and c-di-GMP serves as an allosteric activator of the BcsA subunit of cellulose synthase (19). The mechanisms linking motility, biofilm formation, and oxygen remain poorly understood. These findings suggest that oxygen signaling and associated c-di-GMP dynamics, mediated by oxygen-responsive DGCs or PDEs, play a key role in regulating the physiological state of FY-07.

In this study, we reveal that the endophytic bacterium *K. oryzendophytica* FY-07 senses oxygen via an antagonistic DGC-PDE pair containing PAS domains and modulates intracellular c-di-GMP levels with opposing effects to balance swarming motility and BC production. Unlike previously reported oxygen-sensing DGC/PDE systems, this module directly links oxygen perception to cellulose biosynthesis, uncovering a novel mechanism underlying oxygen-mediated lifestyle transitions. These findings provide new insights into the ecological adaptation and regulatory strategies of *Kosakonia* species and offer potential guidance for optimizing BC production and controlling biofilm formation.

## RESULTS AND DISCUSSION

### Domain architectures of c-di-GMP metabolic enzymes encoded near the cellulose synthase operon

**Fig 1:**
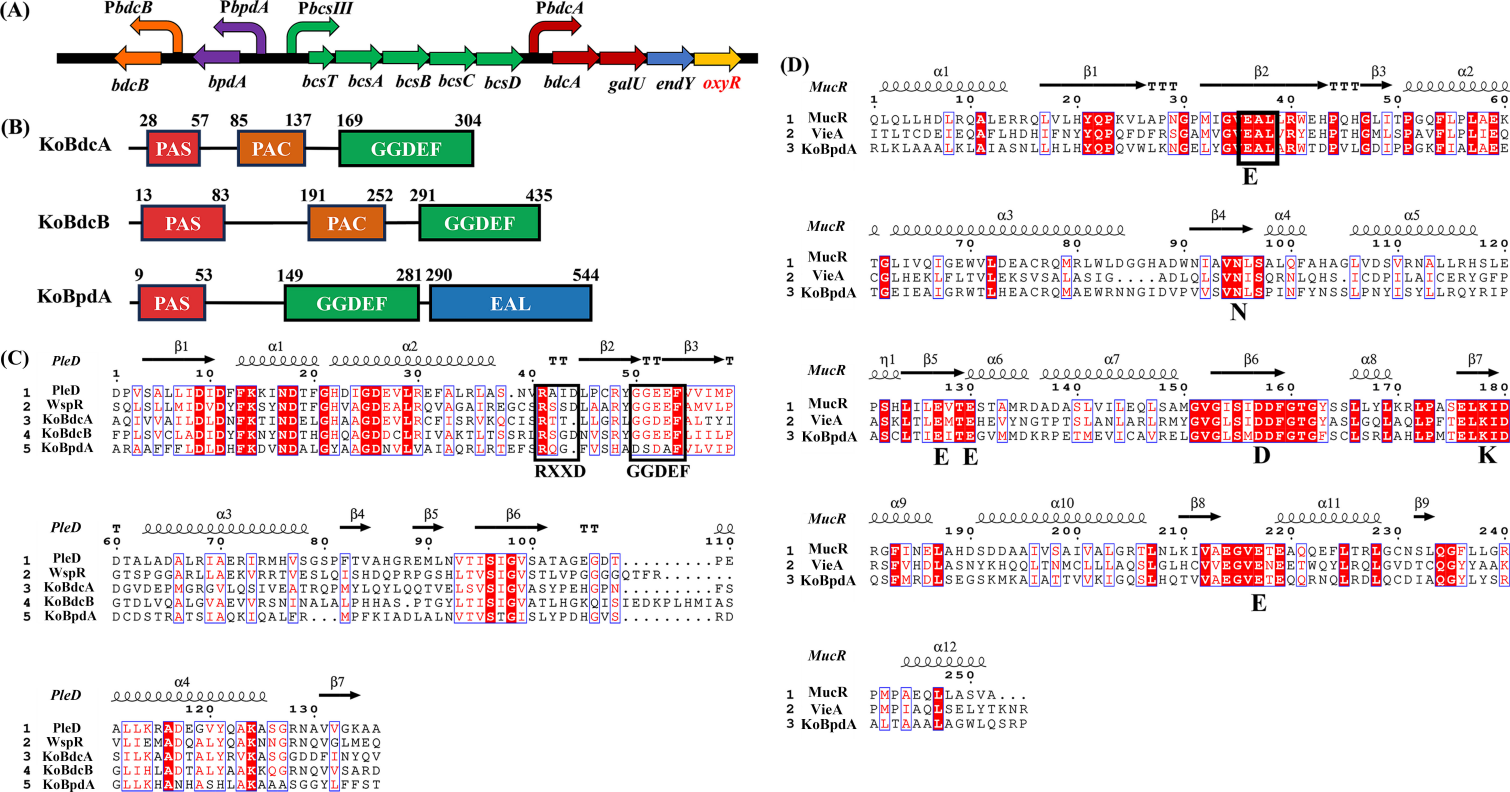
(**A**) Genetic organization of the bcsIII operon. (**B**) Domain architectures of KoBdcA, KoBdcB, and KoBpdA were predicted using the UniProt database. (**C**) Sequence alignment of the GGDEF domain with well-characterized DGCs PleD and WspR. (**D**) Sequence alignment of the EAL domain with well-characterized PDEs MucR and VieA. The catalytic motifs of the DGC domain (I-site “RXXD” and A-site “GG(D/E)EF”) and the PDE domain (“EAL” and “ENEEDKE” motif) are highlighted with black boxes.

Gene annotation of *K. oryzendophytica* FY-07 (GenBank accession no. CP012487.1) was performed using the National Center for Biotechnology Information (NCBI) Prokaryotic Genome Annotation Pipeline. Conserved domain searches with the NCBI Conserved Domain Database identified 53 putative DGCs and PDEs containing GGDEF, EAL, or HD-GYP motifs, reflecting the complexity of its c-di-GMP signaling system (20). To elucidate the regulatory mechanism of BC production, we specifically examined genes closely associated with cellulose synthesis. The cellulose synthase operon III (*bcsIII*), primarily responsible for BC biosynthesis in *K. oryzendophytica* FY-07, comprises the genes *bcsT*, *bcsA*, *bcsB*, *bcsC*, and *bcsD* (Fig. 1A) (21). Based on gene annotation and domain prediction using the UniProt database (https://www.uniprot.org/) (22), three proteins located adjacent to the *bcsIII* operon were identified as potentially involved in c-di-GMP metabolism. In *K. oryzendophytica* FY-07, we designated AKI40_0890 as KoBdcA (*bcsIII* operon-adjacent diguanylate cyclase A), AKI40_0897 as KoBdcB (*bcsIII* operon-adjacent diguanylate cyclase B), and AKI40_0896 as KoBpdA (*bcsIII* operon-adjacent phosphodiesterase A). KoBdcA and KoBdcB contained PAS, PAC, and GGDEF domains, while KoBpdA possessed PAS, GGDEF, and EAL domains (Fig. 1B). The GGDEF domains of KoBdcA, KoBdcB, and KoBpdA were aligned with those of the well-characterized DGCs PleD (from *Caulobacter vibrioides* NA1000) and WspR (from *Pseudomonas aeruginosa* PAO1). As shown in Fig. 1C, both KoBdcA and KoBdcB contain the conserved active site (A-site) “GG(D/E)EF” motif. KoBdcB also includes the “RXXD” motif, which functions as an inhibitory site (I-site) located upstream of the “GG(D/E)EF” catalytic motif. The I-site facilitates negative feedback regulation of DGC activity, preventing excessive intracellular GTP consumption (23). Proteins containing tandem GGDEF-EAL domains can hydrolyze c-di-GMP or function as bifunctional enzymes that both synthesize and degrade it. KoBpdA lacked the conserved “GG(D/E)EF” motif, suggesting that it may not possess DGC activity. The EAL domain of KoBpdA was aligned with those of the well-characterized PDEs MucR (from *Pseudomonas aeruginosa* PAO1) and VieA (from *Vibrio cholerae* serotype O1). As shown in Fig. 1D, KoBpdA contains the conserved “EAL” motif and seven key residues (ENEEDKE) critical for the catalytic activity of EAL domains. These findings suggest that KoBpdA likely functions solely as a PDE. In most cases, such GGDEF-EAL dual-domain proteins exhibit only one catalytic activity, while the other domain may serve a structural or regulatory role in modulating overall protein function (24).

### *In vitro* functional characterization of KoBdcA, KoBdcB, and KoBpdA

**Fig 2:**
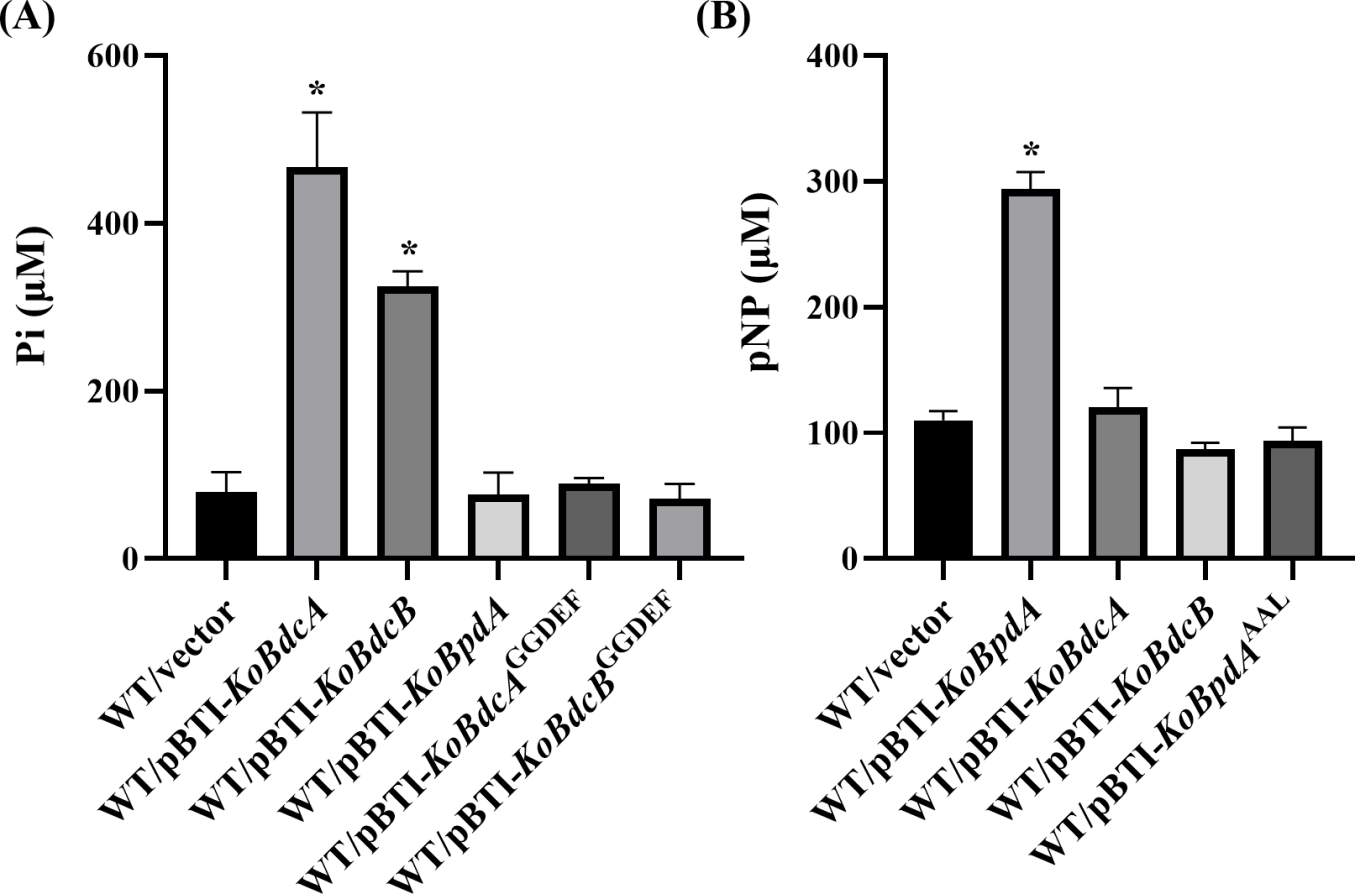
(**A**) Pi concentration in the DGC assay. DGC activity was determined by measuring PPi release from GTP using the EnzChek Pyrophosphate Assay Kit, with detection at 360 nm. (**B**) pNP concentration in the PDE assay. PDE activity was determined using bis-pNPP as the substrate, with p-nitrophenol release quantified at 410 nm after incubation at 37°C for 60 min. *P < 0.05.

Domain analysis indicated that KoBdcA and KoBdcB likely possess DGC activity, while KoBpdA appears to exhibit PDE activity. To test these hypotheses, we overexpressed *KoBdcA*, *KoBdcB*, and *KoBpdA* under the control of an isopropyl β-D-1-thiogalactopyranoside (IPTG)-inducible promoter in the wild-type (WT) FY-07 strain using plasmids pBTI-*KoBdcA*, pBTI-*KoBdcB*, and pBTI-*KoBpdA*, respectively. DGC activity was quantified using a coupled spectrophotometric assay that measures the release of inorganic pyrophosphate (PPi), a byproduct of the DGC-catalyzed synthesis of c-di-GMP (25). Because direct detection of PPi is challenging, it was enzymatically converted into two molecules of inorganic phosphate (Pi), and the Pi standard curve is shown in Fig. S1A. As shown in Fig. 2A, both wild-type WT/pBTI-*KoBdcA* and WT/pBTI-*KoBdcB* exhibited significantly higher Pi levels than WT/vector, confirming that KoBdcA and KoBdcB function as DGCs. In contrast, no significant difference in Pi release was observed between WT/pBTI-*KoBpdA* and WT/vector, likely due to the degenerate GGDEF domain in KoBpdA, which renders it catalytically inactive. DGC activity requires the dimerization of two identical GGDEF domains to bind GTP and generate c-di-GMP, a process often regulated by auxiliary domains (26, 27). Therefore, the N-terminal PAS domains of KoBdcA and KoBdcB may facilitate proper protein oligomerization, enabling the formation of catalytically active enzymes (28). Indeed, deletion of the N-terminal PAS domains (WT/pBTI-*KoBdcA*^GGDEF^ and WT/pBTI-*KoBdcB*^GGDEF^) abolished their DGC activity.

PDE activity was evaluated using a colorimetric assay based on the hydrolysis of the synthetic substrate bis-p-nitrophenyl phosphate (bis-pNPP). In the presence of enzymatic PDE activity, bis-pNPP is cleaved at its phosphodiester bond to release p-nitrophenol (pNP), which can be quantitatively measured (29). The standard curve for pNP quantification is shown in Fig. S1B. As shown in Fig. 2B, WT/pBTI-*KoBpdA* exhibited significantly higher PDE activity than WT/vector, indicating that KoBpdA functions as an active PDE. In contrast, no increase in PDE activity was detected in WT/pBTI-*KoBdcA* or WT/pBTI-*KoBdcB*. Furthermore, a point mutant (WT/pBTI-KoBpdA^AAL^), in which the EAL motif was substituted with AAL, completely abolished PDE activity, suggesting that the conserved EAL motif is essential for the enzymatic function of KoBpdA.

### Effects of KoBdcA, KoBdcB, and KoBpdA on BC production and intracellular c-di-GMP levels

FY-07 is capable of producing the exopolysaccharide BC. The red, dry, and rough (rdar) colony morphology is indicative of its biofilm-forming ability and varies phenotypically according to its BC production capacity (30). The effects of KoBdcA, KoBdcB, and KoBpdA on BC production and rdar colony morphology in FY-07 were evaluated using single- and double-gene deletion mutants, their corresponding complementation strains, and overexpression strains. Figure S2 shows that all strains grew similarly under aerobic, microaerobic, and anaerobic conditions, indicating that the knockouts, complementation, and overexpression did not affect growth.

**Fig 3:**
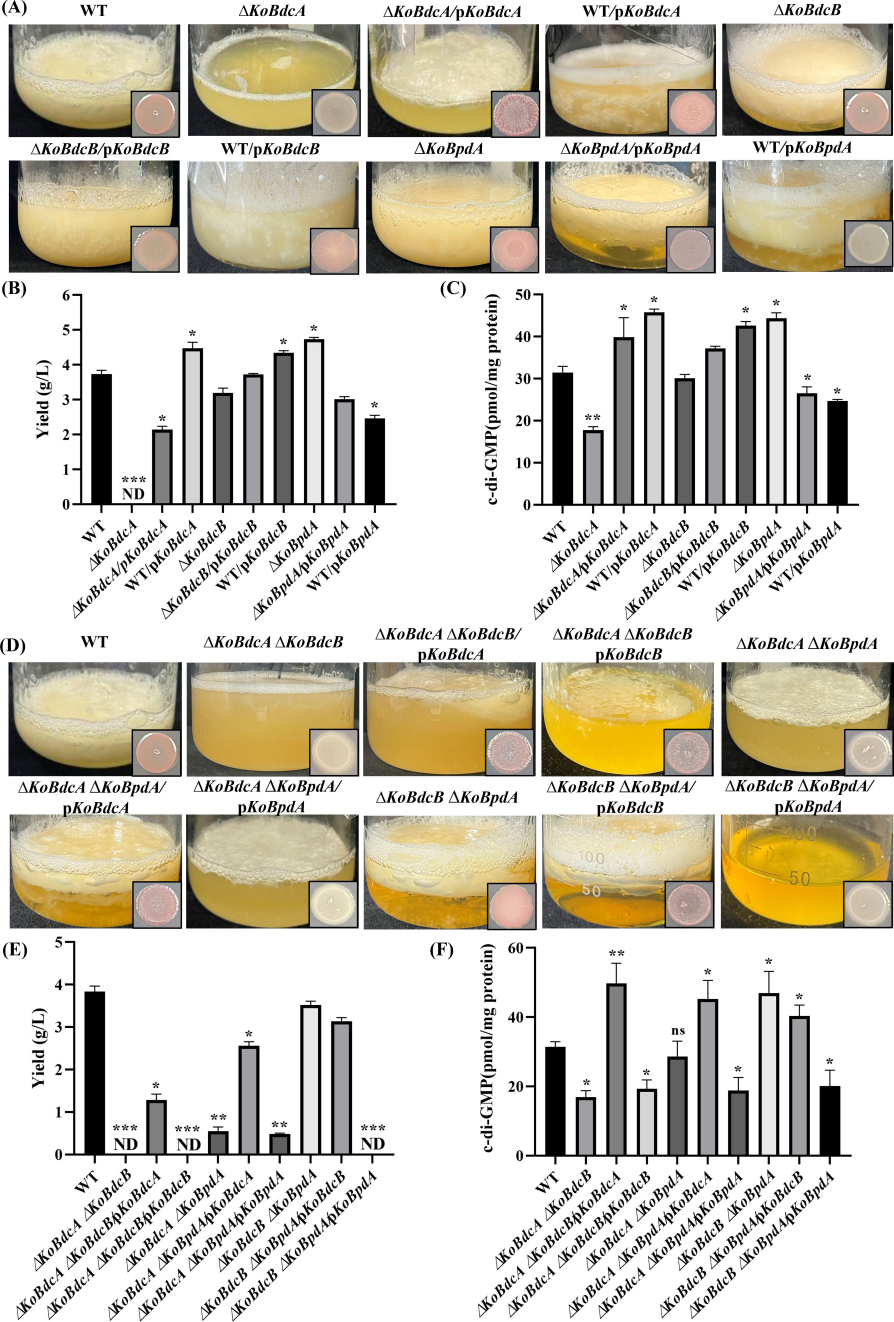
(**A**) Fermentation and rdar colony morphology, (**B**) BC yield, and (**C**) intracellular c-di-GMP levels of WT, knockout, complemented, and overexpression strains. (**D**) Fermentation and rdar morphology, (**E**) BC yield, and (**F**) intracellular c-di-GMP levels of WT, double knockout, and complemented strains. c-di-GMP was extracted from whole cells, and levels were measured using HPLC. For direct comparison, the same WT strain was used as the control for both single and double knockout mutants. *P < 0.05; **P < 0.01; ***P < 0.001; and ns, no significance.

BC from all strains was collected, purified by alkali treatment and water washing to neutrality, and then lyophilized to constant weight for yield determination. In BC-producing strains, cellulose fibers create a wrinkled colony surface on Congo Red plates, where the dye binds to the β-1,4-glucans of BC, rendering cellulose-containing colonies red (31). As shown in Fig. 3A, FY-07 WT produces BC at 3.77 g/L with characteristic red, smooth colonies. BC production is abolished in ∆*KoBdcA*, partially restored in the complementation strain, and increased in the overexpression strain (4.47 g/L), correlating with altered colony morphologies. The enhanced BC yield likely results from elevated c-di-GMP levels activating the cellulose synthase complex (32). In contrast, ∆*KoBdcB* and ∆*KoBdcB*/p*KoBdcB* strains do not significantly differ from WT in BC production. However, WT/p*KoBdcB* shows a slight increase in BC yield (4.34 g/L), with colonies exhibiting full-surface wrinkling. The ∆*KoBpdA* mutant produces red, fully wrinkled colonies with a high BC yield of 4.73 g/L. Conversely, BC production in ∆*KoBpdA*/p*KoBpdA* and WT/p*KoBpdA* is significantly reduced (3.01 g/L and 2.46 g/L, respectively), with colonies displaying light pink to white coloration. The high-copy plasmid-mediated overexpression of *KoBpdA* likely causes excessive PDE activity, reducing intracellular c-di-GMP levels and consequently decreasing cellulose synthesis. These findings suggest that the three enzymes exert distinct regulatory effects on BC production and rdar colony morphology in FY-07.

Given the roles of DGC- and PDE-encoded enzymes in c-di-GMP metabolism, the impact of KoBdcA, KoBdcB, and KoBpdA on intracellular c-di-GMP concentrations is evaluated using high-performance liquid chromatography (HPLC). As shown in Fig. 3C, the intracellular c-di-GMP level in the WT strain is 31.42 pmol/mg protein. The ∆*KoBdcA* mutant shows a significant reduction to 17.76 pmol/mg protein, likely because KoBdcA functions as a DGC, and its absence decreases c-di-GMP synthesis. Due to the high copy number of the expression plasmid, both ∆*KoBdcA*/p*KoBdcA* and WT/p*KoBdcA* exhibited significantly elevated c-di-GMP levels (39.84 and 45.76 pmol/mg protein, respectively). In contrast, neither ∆*KoBdcB* nor ∆*KoBdcB*/p*KoBdcB* shows a significant change in c-di-GMP levels, although WT/p*KoBdcB* displays a moderate increase. This result may reflect functional redundancy among DGC-encoding enzymes or indicate that the deleted DGC is active only within specific subcellular regions, thus exerting limited effects on total c-di-GMP concentrations (12, 33). Furthermore, c-di-GMP signaling is known to be target-specific, with certain DGCs or PDEs directing c-di-GMP to distinct effectors (34, 35). Therefore, KoBdcB might act as a spatially restricted DGC or lack specificity for cellulose synthase. The ∆*KoBpdA* mutant exhibits a significant increase in c-di-GMP concentration to 44.40 pmol/mg protein, indicating that KoBpdA acts as a PDE whose loss leads to intracellular c-di-GMP accumulation. Overexpression of KoBpdA in ∆*KoBpdA*/p*KoBpdA* and WT/p*KoBpdA* significantly reduces c-di-GMP levels, consistent with enhanced PDE activity. Collectively, these findings indicate that KoBdcA and KoBpdA effectively regulate BC synthesis in FY-07, with KoBdcA primarily promoting and KoBpdA predominantly inhibiting BC production through modulation of intracellular cyclic di-GMP levels. In contrast, KoBdcB exhibits weak catalytic activity.

Combinatorial gene deletion analysis was performed to further elucidate the roles of these enzymes within the regulatory network. As shown in Fig. 3D, the ∆*KoBdcA* ∆*KoBdcB* double mutant exhibited a complete loss of BC production. Complementation with KoBdcA partially restored this phenotype, whereas complementation with KoBdcB had minimal effect. Colony morphology and BC yield were consistent with the fermentation results. Intracellular c-di-GMP quantification revealed a significant reduction in the double mutant, which was not reversed by expressing KoBdcB alone. The intracellular c-di-GMP levels in the ∆*KoBdcA* ∆*KoBdcB* mutant and the ∆*KoBdcA* ∆*KoBdcB*/p*KoBdcB* strain were 16.94 and 19.33 pmol/mg protein, respectively. In contrast, ∆*KoBdcA* ∆*KoBdcB*/p*KoBdcA* showed a significant increase to 49.76 pmol/mg protein; however, this was insufficient to fully restore BC synthesis. In the ∆*KoBdcA* ∆*KoBpdA* mutant, BC production was significantly reduced but not entirely abolished. Complementation with KoBdcA (∆*KoBdcA* ∆*KoBpdA*/p*KoBdcA*) effectively rescued the BC synthesis phenotype, indicating that KoBdcA requires KoBdcB for full functional activity in activating cellulose biosynthesis. However, complementation with KoBpdA (∆*KoBdcA* ∆*KoBpdA*/p*KoBpdA*) had no significant effect. Colony morphology, BC yield, and fermentation data supported these findings. Intracellular c-di-GMP levels in the ∆*KoBdcA* ∆*KoBpdA* mutant were comparable with those in the WT strain, indicating a mutual compensatory effect upon dual deletion and supporting the hypothesis that KoBdcA and KoBpdA act antagonistically. Specifically, ∆*KoBdcA* ∆*KoBpdA*/p*KoBdcA* exhibited a significant increase in intracellular c-di-GMP and a corresponding restoration of BC production, whereas ∆*KoBdcA* ∆*KoBpdA*/p*KoBpdA* showed a significant reduction in c-di-GMP and failed to rescue BC synthesis. In the ∆*KoBdcB* ∆*KoBpdA* double mutant, BC production showed a mild but statistically insignificant decrease. Under this double knockout background, complementation with KoBdcB did not restore the phenotype, whereas reintroduction of KoBpdA completely abolished BC production. Notably, intracellular c-di-GMP levels in ∆*KoBdcB* ∆*KoBpdA* were significantly elevated to 46.97 pmol/mg protein. Complementation with KoBdcB (∆*KoBdcB* ∆*KoBpdA*/p*KoBdcB*) had little effect, whereas reintroduction of KoBpdA (∆*KoBdcB* ∆*KoBpdA*/p*KoBpdA*) significantly reduced the c-di-GMP concentration to 20.15 pmol/mg protein.

### Effects of KoBdcA, KoBdcB, and KoBpdA on the motility of FY-07

**Fig 4:**
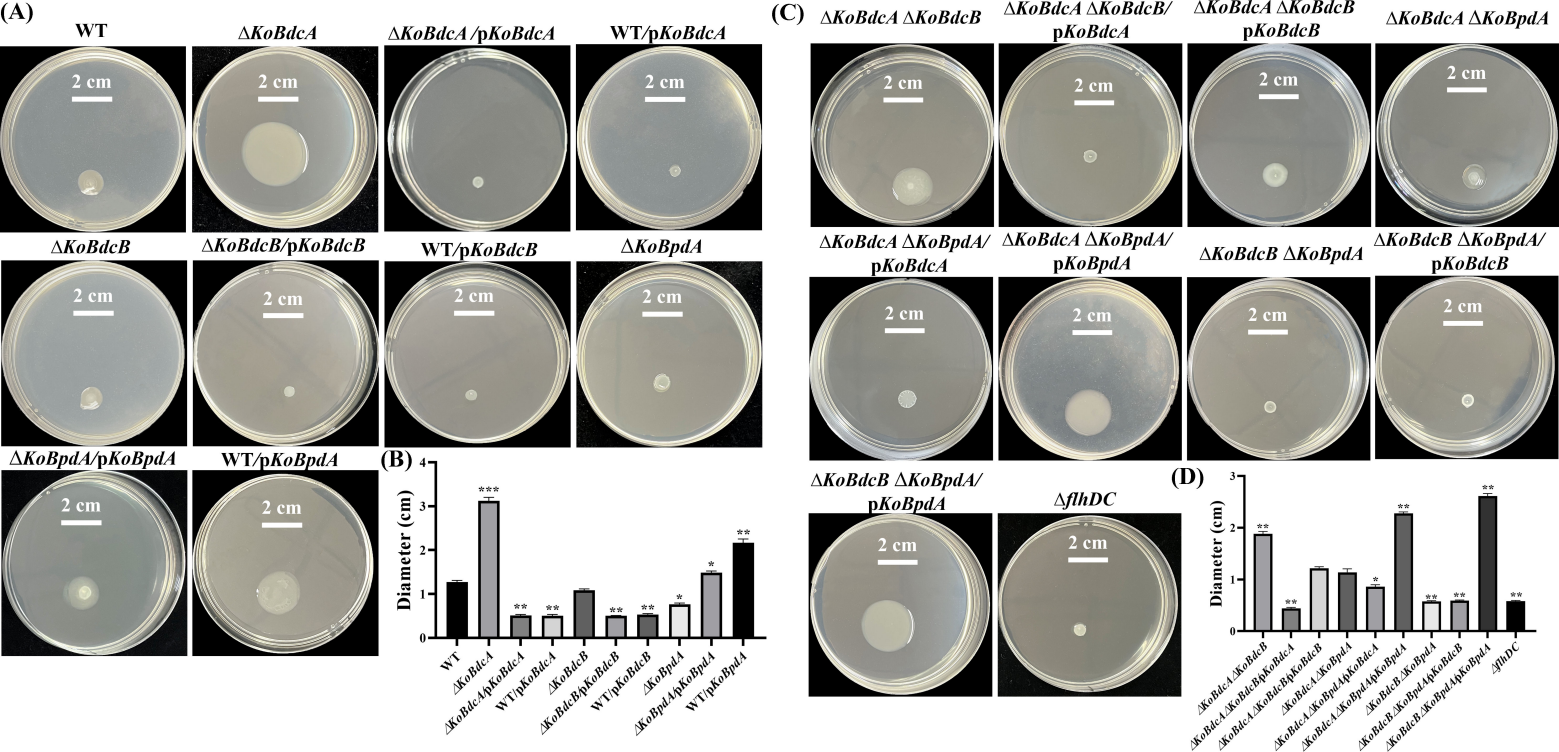
(**A**) Swarming motility of bacterial strains. (**A and B**) representative images and measured swarm diameters of wild-type, single-knockout, and overexpression strains; (**C and D**) representative images and measured swarm diameters of wild-type, double-knockout, and complementation strains. For direct comparison, the same WT strain was used as the control for both single and double knockout mutants. *P < 0.05; **P < 0.01; ***P < 0.001.

c-di-GMP critically regulates the transition between motile and sessile states, enabling bacteria to adapt to environmental changes (36). Therefore, investigating whether intracellular c-di-GMP levels influence the motility of FY-07 was of critical importance. Swimming is flagella-driven individual motility in liquid, largely independent of surface or extracellular factors, whereas swarming is a collective movement on semi-solid surfaces that depends on extracellular polysaccharides, surfactants, and cell-surface adhesion (36, 37). The ∆*KoBdcA*, ∆*KoBdcB*, and ∆*KoBpdA* mutants exhibited swimming motility comparable with that of the FY-07 WT strain (Fig. S3). Consequently, c-di-GMP regulates FY-07 motility predominantly through its effects on swarming behavior. As shown in Fig. 4A, Δ*KoBdcA* significantly enhanced swarming motility, with an average swarming diameter of 3.11 cm. In contrast, Δ*KoBdcA*/p*KoBdcA* and WT/p*KoBdcA* showed significantly reduced swarming, consistent with intracellular c-di-GMP levels, which were significantly reduced in Δ*KoBdcA* and elevated following KoBdcA complementation and overexpression. Δ*KoBdcB* did not significantly affect swarming compared with the WT; however, both Δ*KoBdcB*/p*KoBdcB* and WT/p*KoBdcB* exhibited decreased swarming motility. Intracellular c-di-GMP levels were slightly elevated in Δ*KoBdcB*/p*KoBdcB* relative to WT; however, the difference was not statistically significant. Nevertheless, the observed suppression of swarming may be attributed to the high sensitivity of swarming behavior to even slight increases in c-di-GMP levels upon DGC overexpression. Swarming motility was significantly diminished in the Δ*KoBpdA* mutant, accompanied by a significant elevation in intracellular c-di-GMP levels. Overexpression of the phosphodiesterase KoBpdA in Δ*KoBpdA*/p*KoBpdA* and WT/p*KoBpdA* strains increased swarming diameters, possibly due to the reduction in c-di-GMP levels, which suppressed biofilm formation and promoted a planktonic state favorable for bacterial dispersal (37). Collectively, these findings demonstrate that BC synthesis and motility are antagonistic processes in FY-07, with state transitions mediated by c-di-GMP. KoBdcA and KoBpdA play major roles in regulating these processes.

Swarming motility was also assessed in the double knockout strains. As shown in Fig. 4C, the ∆*KoBdcA* ∆*KoBdcB* strain exhibited significantly enhanced motility. Complementation with KoBdcA (∆*KoBdcA* ∆*KoBdcB*/p*KoBdcA*) significantly reduced motility, while complementation with KoBdcB (∆*KoBdcA* ∆*KoBdcB*/p*KoBdcB*) restored motility to WT levels. The ∆*KoBdcA* ∆*KoBpdA* mutant strains exhibited motility comparable with that of the WT strain. Notably, KoBpdA complementation (∆*KoBdcA* ∆*KoBpdA*/p*KoBpdA*) significantly increased motility, whereas KoBdcA complementation (∆*KoBdcA* ∆*KoBpdA*/p*KoBdcA*) caused a slight reduction. In contrast, the ∆*KoBdcB* ∆*KoBpdA* double knockout strain showed a pronounced decrease in motility. Complementation with KoBdcB (∆*KoBdcB* ∆*KoBpdA*/p*KoBdcB*) alone failed to restore the phenotype, whereas KoBpdA complementation (∆*KoBdcB* ∆*KoBpdA*/p*KoBpdA*) significantly enhanced swarming motility. The FlhDC complex serves as the master regulator of flagellar biosynthesis in diverse bacterial species, including *Escherichia coli*, *Salmonella*, and *Kosakonia* (38–40). Disruption of the flhDC genes in FY-07 abolished collective motility under conditions where flagellar function was compromised (Fig. 4C). This finding demonstrates that the observed motility is primarily driven by flagella-dependent swarming rather than passive diffusion or twitching motility, thereby validating the swarming phenotype of FY-07 on agar plates.

These results indicated that *KoBdcA* and *KoBpdA* functioned as antagonistic regulators: *KoBdcA* catalyzed the synthesis of c-di-GMP, thereby promoting BC production and inhibiting swarming motility, while *KoBpdA* facilitated c-di-GMP degradation, suppressing BC synthesis and enhancing motility. The double deletion of these genes had minimal impact on overall intracellular c-di-GMP levels. This may result from other DGC-PDE pairs in FY-07 compensating for the loss of KoBdcA/KoBpdA to maintain global c-di-GMP homeostasis or from KoBdcA/KoBpdA acting locally at specific sites (e.g., cellulose synthase or flagellar operons). In contrast, deleting either gene significantly altered intracellular c-di-GMP. KoBdcB showed weaker activity, slightly affecting BC production and motility, but was required for full KoBdcA-mediated BC synthesis, suggesting a mediating or partial catalytic role.

### Interactions between KoBdcA, KoBdcB, and KoBpdA proteins

**Fig 5:**
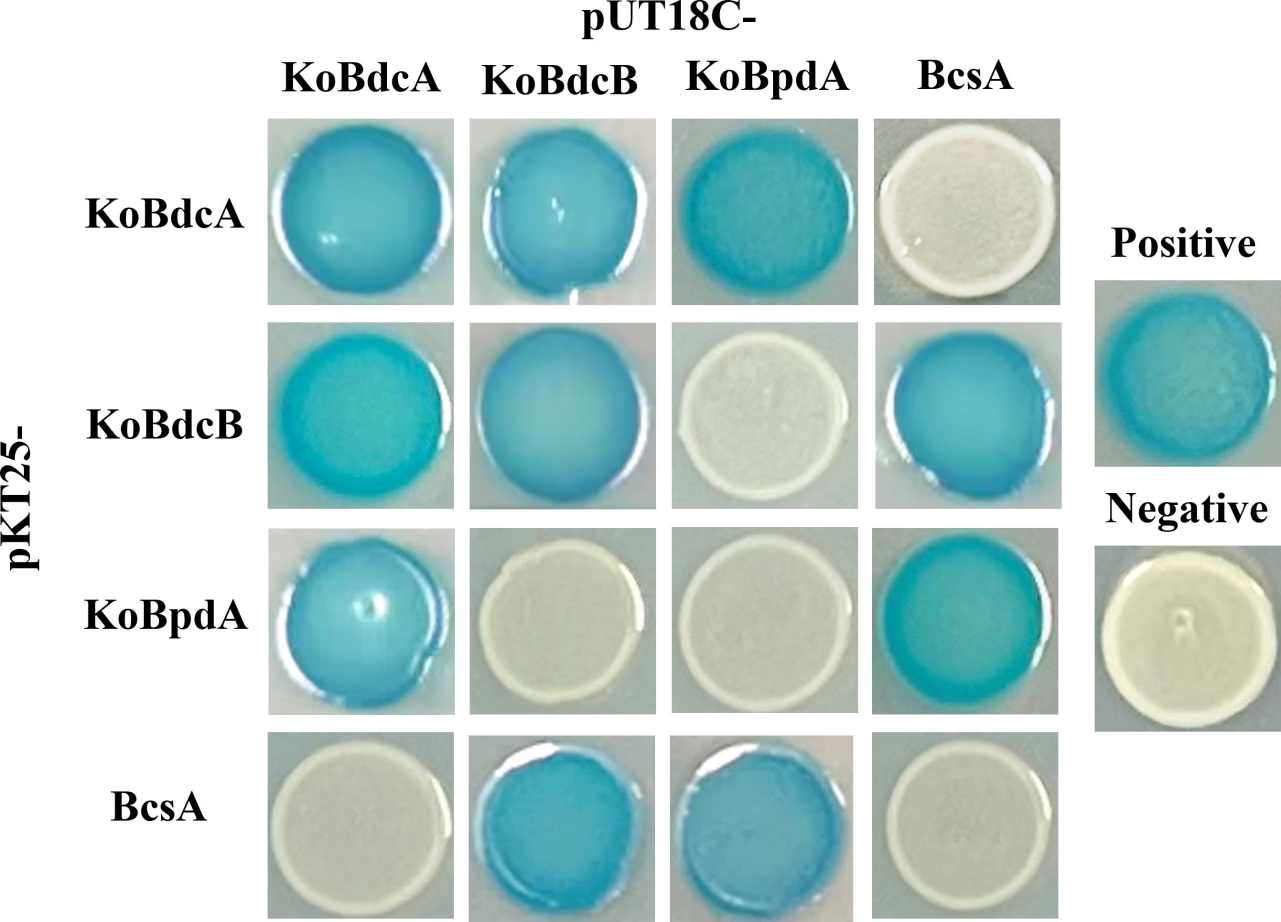
Interactions between KoBdcA, KoBdcB, KoBpdA, and the cellulose synthase subunit BcsA were analyzed using a bacterial two-hybrid system.

These findings indicate that KoBdcA and KoBpdA have opposing activities and phenotypes. Their genomic proximity and PAS domains suggest potential interaction, as PAS domains often mediate protein interactions and GGDEF domains act via dimerization (41). These interactions were investigated using bacterial adenylate cyclase two-hybrid (BACTH) assays. As shown in Fig. 5, bidirectional interaction assays were conducted among KoBdcA, KoBdcB, and KoBpdA, as well as with BcsA, the catalytic subunit of the cellulose synthase complex. KoBdcA exhibited self-interaction, confirming its ability to dimerize (42) and also interacted directly with both KoBdcB and KoBpdA, but not with BcsA. In contrast, KoBdcB interacted directly with KoBdcA, BcsA, and itself, but not with KoBpdA, suggesting that KoBdcB possesses dimerization potential and may associate with the cellulose synthase complex. KoBpdA interacted with KoBdcA and BcsA, but not with KoBdcB or itself. Unlike DGCs, which require homodimer formation to catalyze the condensation of two GTP molecules, such dimerization appears unnecessary for the catalytic activity of PDEs (36).

PAS domains are widely recognized as critical modules that mediate protein-protein interactions (43). To clarify their roles, we examined the PAS domains of KoBdcA, KoBdcB, and KoBpdA in mediating intermolecular interactions (Fig. S4). The interactions of isolated PAS domains mirror those of the full-length proteins. The PAS domain of KoBdcA self-associates and interacts with KoBdcB and KoBpdA; KoBdcB’s PAS domain interacts with itself, KoBdcA, and BcsA; and KoBpdA’s PAS domain interacts with KoBdcA and BcsA. Removal of PAS domains abolished all detectable interactions, whereas GGDEF or EAL domains showed none, indicating that PAS domains mediate these interactions. Together, these results suggest that KoBdcA interacts with BcsA indirectly via KoBdcB, while KoBpdA interacts directly with BcsA, forming a ternary DGC-PDE module near the cellulose synthase complex that dynamically regulates local c-di-GMP levels (44).

### Oxygen functions as the key signaling molecule in the KoBdcA/KoBdcB/KoBpdA ternary system

**Fig 6:**
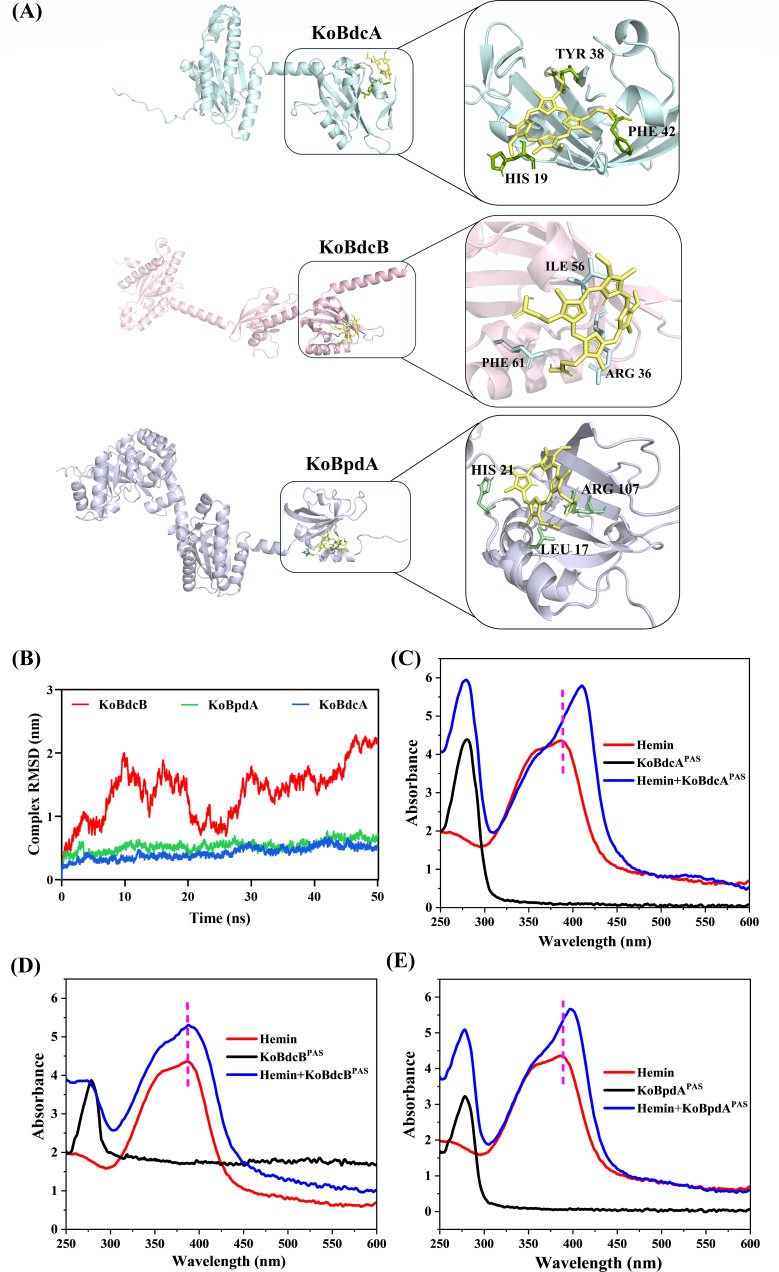
(**A**) Molecular docking results of KoBdcA, KoBdcB, and KoBpdA with the hemin molecule. (**B**) RMSD values of the KoBdcA-, KoBdcB-, and KoBpdA-hemin complexes over a 50 ns molecular dynamics simulation. (**C–E**) UV-Vis spectra of the PAS domain proteins of KoBdcA, KoBdcB, and KoBpdA in the presence of hemin.

UniProt analysis predicts a heme-binding pocket in the PAS domain of KoBdcA, suggesting oxygen sensing via hemin binding. We therefore examined hemin binding in the PAS domains of all three proteins (45). As all three are membrane proteins that are difficult to purify, we first use AlphaFold for structural prediction and perform molecular docking to assess their hemin-binding capacity. As shown in Fig. 6A, molecular docking reveals that KoBdcA and KoBpdA have strong binding affinities for hemin, while KoBdcB shows weaker binding potential. KoBdcA interacts with hemin via HIS19, TYR38, and PHE42, forming stable π–π stacking and hydrogen bonds (46). KoBpdA binds hemin through HIS21, ARG107, and LEU17 in a similarly stable manner. In contrast, KoBdcB involves ARG36, ILE56, and PHE61, showing a less favorable binding mode. These results are supported by molecular dynamics (MD) simulations (Fig. 6B), where the root mean square deviation (RMSD) values of KoBdcA-hemin and KoBpdA-hemin complexes remain below 1.0 nm over 50 ns, suggesting good structural stability. The KoBdcB-hemin complex shows large RMSD fluctuations (>2.5 nm), indicating poor stability and conformational instability.

The PAS domains of KoBdcA, KoBdcB, and KoBpdA are purified and incubated with hemin. UV-Vis spectra (Fig. 6C through E) show that all three proteins exhibit absorption peaks at 280 nm. The Soret band of hemin (~385–405 nm) redshifts and intensifies significantly with KoBdcA^PAS^ and KoBpdA^PAS^, indicating strong binding. In contrast, KoBdcB^PAS^ shows a weaker absorbance change, suggesting a weak interaction. These findings demonstrate that KoBdcA and KoBpdA are capable of binding hemin, whereas KoBdcB interacts exclusively with KoBdcA and not with hemin, likely due to structural differences in their PAS domains. Hemin, an iron-containing metalloporphyrin that reversibly binds oxygen, has been widely reported to function as a prosthetic group, enabling PAS domain proteins to sense oxygen (47, 48). Accordingly, oxygen signaling is likely a key regulatory factor modulating the activity of the KoBdcA-KoBpdA “DGC-PDE” pair.

**Fig 7:**
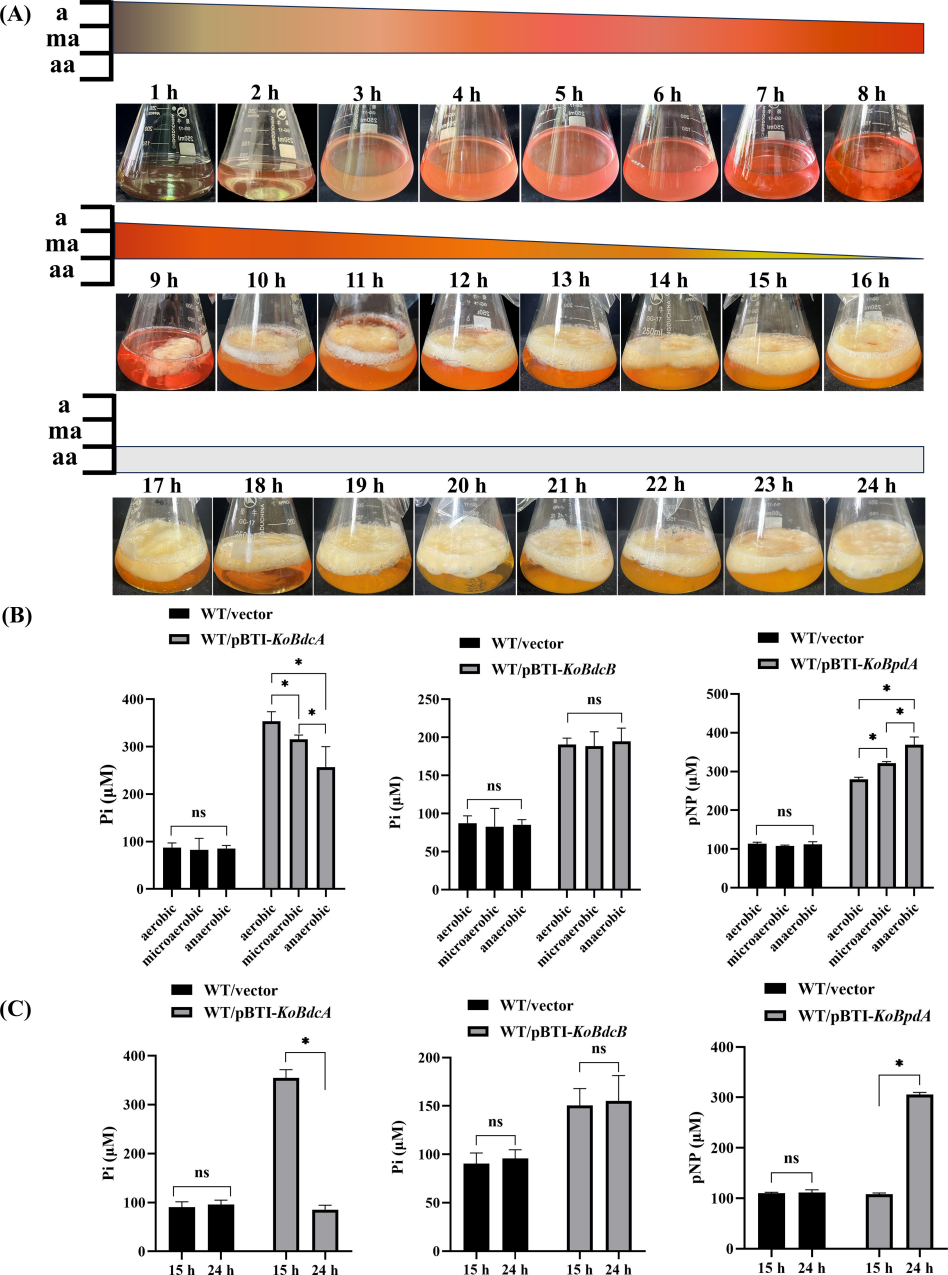
(**A**) Fermentation images of FY-07 from 1 to 24 h in medium supplemented with resazurin as an oxygen indicator under aerobic conditions. a: aerobic; ma: microaerobic; and aa: anaerobic. (**B**) Enzymatic activities of KoBdcA, KoBdcB, and KoBpdA as DGCs or PDEs under aerobic, microaerobic, and anaerobic conditions. (**C**) Comparison of DGC or PDE enzymatic activities of KoBdcA, KoBdcB, and KoBpdA at 15 h and 24 h of fermentation. Data are presented as mean ± SD (*n* = X). Statistical significance was determined by one-way ANOVA, followed by a post-hoc test. *P < 0.05 compared with WT; ns, no significance.

To examine oxygen signaling in the KoBdcA-KoBpdA DGC-PDE pair, resazurin (1 g•L^–1^) was added to aerobic FY-07 cultures, and color changes were recorded hourly (Fig. 7A). The solution turned from dark blue to blue-green within 1 h, indicating high oxygen, then pink after 2 h as oxygen decreased. From 2 to 8 h (aerobic to microaerobic), it deepened to dark red, reflecting predominance of oxidized resazurin. From 9 to 16 h, the solution gradually turned orange-red with BC pellicle formation, reflecting oxygen consumption and partial resazurin reduction. Between 17 and 24 h, it became orange-yellow, as resazurin was nearly fully reduced and BC synthesis ceased. The medium cleared until ~20 h, then turbidity appeared by 21 h and peaked at 24 h, indicating that FY-07 entered a motile, proliferative state.

FY-07 fermentation gradually shifted from aerobic to microaerobic and then anaerobic. In the late stage (17–24 h), the lower layer became turbid as BC production ceased, reflecting a metabolic switch to motile proliferation likely regulated by oxygen. PAS domains sense oxygen via heme binding and conformational changes, modulating downstream pathways (43, 49). Since both KoBdcA and KoBpdA were capable of binding hemin, we assessed their catalytic activities under aerobic, microaerobic, and anaerobic conditions (Fig. 7B). Compared with the WT/vector strain, overexpression of KoBdcA (WT/pBTI-*KoBdcA*) resulted in a gradual decrease in DGC activity as oxygen levels declined from aerobic to anaerobic conditions. In contrast, overexpression of KoBdcB (WT/pBTI-*KoBdcB*) showed no significant differences in DGC activity under varying oxygen conditions, consistent with its inability to bind hemin and suggesting that its catalytic activity was independent of oxygen availability. When KoBpdA was overexpressed (WT/pBTI-*KoBpdA*), its PDE activity increased as oxygen levels decreased, exhibiting the opposite trend to KoBdcA. These results show that oxygen strongly modulates KoBdcA and KoBpdA activities: under oxygen-rich conditions, KoBdcA displayed high DGC activity while KoBpdA was weak, whereas oxygen depletion reduced KoBdcA activity and enhanced KoBpdA activity. Enzymatic assays at 15 h and 24 h revealed stable activity of KoBdcB, higher KoBdcA activity at 15 h, and higher KoBpdA activity at 24 h. This pattern aligns with the shift of FY-07 from aerobic BC production to anaerobic motile proliferation. Enzymatic activities in knockout strains under different oxygen conditions are shown in Fig. S5. The results showed that gene deletions produced opposite trends, further confirming that the KoBdcA-KoBpdA-KoBdcB module responds dynamically to oxygen availability.

Literature and molecular docking analyses have shown that the histidine residue within the heme pocket is essential for hemin binding (45, 50). To verify this, point mutations were introduced into the PAS domains of KoBdcA and KoBpdA (KoBdcA^PASH19A^ and KoBpdA^PASH21A^). The mutant proteins were expressed, purified, and incubated with hemin. UV-Vis spectra (Fig. S6A and B) revealed that both mutants lost their hemin-binding capacity. The corresponding mutant constructs were subsequently overexpressed in the WT background (WT/pBTI-KoBdcA^H19A^ and WT/pBTI-KoBpdA^H21A^). The catalytic activities of these mutants remained unchanged under different oxygen conditions, confirming that hemin binding is crucial for the oxygen-responsive regulation of KoBdcA and KoBpdA (Fig. S6C and D).

The intracellular c-di-GMP levels were monitored hourly during aerobic fermentation (Fig. S7A). From 0 to 5 h, the c-di-GMP levels remained relatively stable, followed by an upward trend beginning at 6 h. Notably, a significant increase in c-di-GMP levels was observed between 7 and 13 h, coinciding with the period of significant BC production. After 14 h, c-di-GMP levels began to decline, and BC production ceased between 17 and 19 h. From 20 to 24 h, c-di-GMP levels decreased significantly, corresponding to the stage when BC was no longer produced and the culture became increasingly turbid due to cell proliferation. Under aerobic, microaerobic, and anaerobic conditions, the intracellular c-di-GMP levels in FY-07 WT remained relatively stable (Fig. S7B), suggesting that additional DGC-PDE pairs contribute to maintaining c-di-GMP homeostasis under low-oxygen conditions.

**Fig 8:**
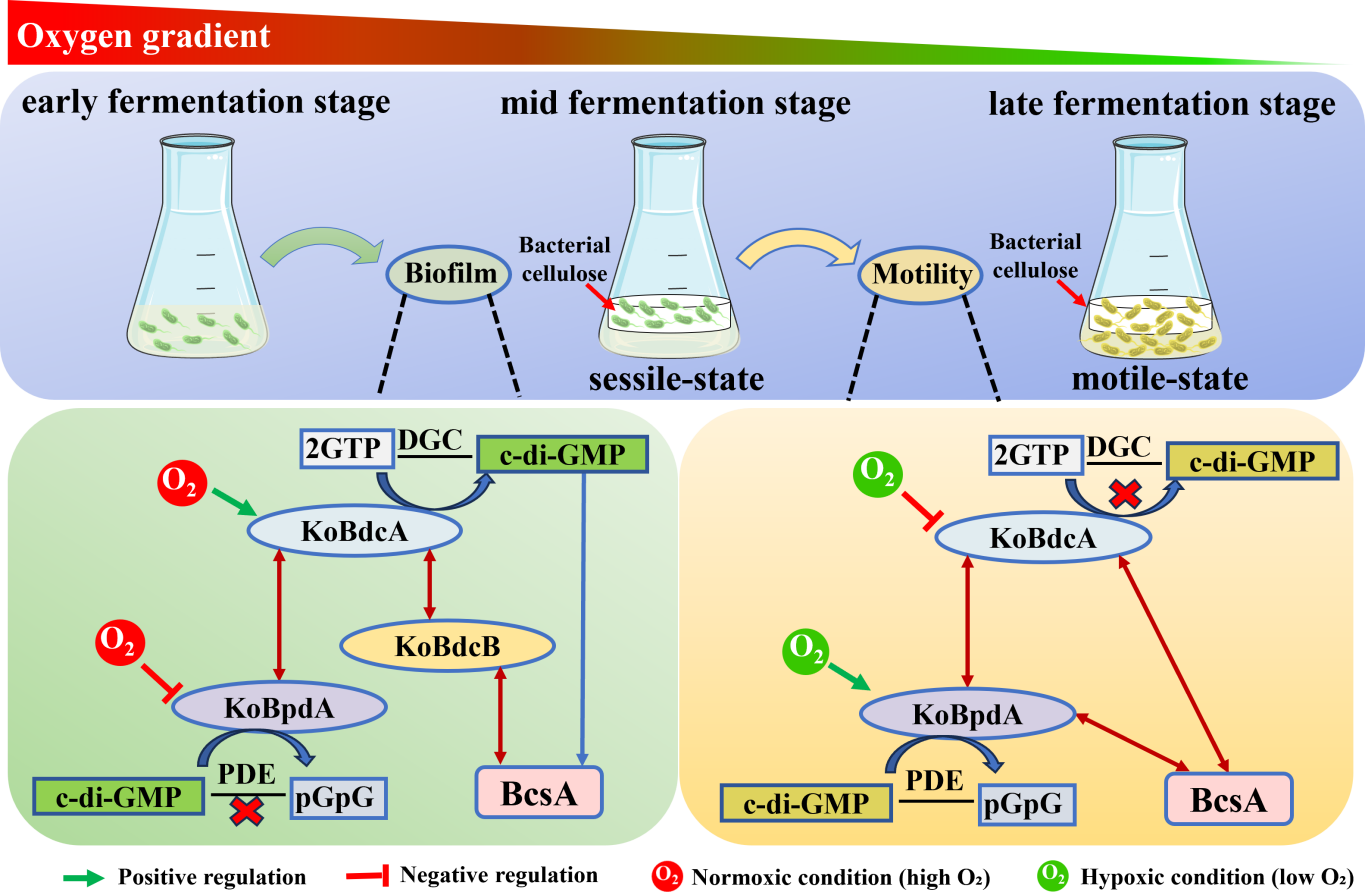
Schematic illustration of how KoBdcA and KoBpdA respond to oxygen signals to regulate c-di-GMP levels, biofilm formation, and motility: FY-07 senses oxygen and produces BC, transitioning to a sessile state during fermentation; at later stages, reduced oxygen induces a return to motility with extensive cell proliferation. Arrows and flat-head lines indicate positive and negative regulation; red and green oxygen denote high and low oxygen levels.

Most previously characterized oxygen-responsive DGC/PDE pairs primarily regulate global c-di-GMP levels, thereby indirectly affecting biofilm formation or motility. For example, *E. coli* DosC/DosP employs PAS-heme domains to sense oxygen and fine-tune intracellular c-di-GMP concentrations (34). Previously reported globin-coupled oxygen sensors (GCSs) also regulate c-di-GMP in response to oxygen; however, each module typically controls only a single enzyme. Representative examples include SA3GCS (globin-coupled DGC) in *Shewanella*, AfGcHK (globin-histidine kinase) in *Anaeromyxobacter*, and YddV/EcDosC (globin-coupled DGC) in *Escherichia coli* (51, 52). These modules generally do not form paired enzyme systems. In contrast, the KoBdcA-KoBpdA-KoBdcB module in *K. oryzendophytica* FY-07 represents a PAS-heme-dependent oxygen sensor directly coupled to the cellulose synthesis operon, forming a localized DGC-PDE network. Hemin-mediated oxygen sensing by KoBdcA and KoBpdA dynamically modulates DGC and PDE activities, enabling reversible transitions between cellulose production and motility. This system provides the direct link between oxygen perception and cellulose biosynthesis, extending oxygen-mediated c-di-GMP regulation from molecular enzymology to the physiological and spatial control of bacterial lifestyle transitions. As shown in Fig. 8, FY-07 employs oxygen-responsive regulation of c-di-GMP metabolism as a key mechanism controlling BC synthesis. The proposed model for KoBdcA and KoBpdA in response to oxygen signaling is as follows: during early aerobic fermentation, high oxygen levels activate KoBdcA as a DGC, catalyzing c-di-GMP synthesis and promoting BC production via KoBdcB-mediated interaction with cellulose synthase BcsA. At the same time, KoBpdA remains inactive or exhibits low activity, keeping cells in a sessile, BC-producing state. From early to mid-fermentation, motile and sessile cells coexist, with sessile cells gradually increasing. As oxygen becomes limited in the mid-to-late fermentation stage, KoBdcA activity declines, while KoBpdA activity rises, reducing intracellular c-di-GMP levels. This decrease weakens c-di-GMP binding to BcsA, halting BC production, while the cells undergo rapid proliferation, indicating a transition to a motile state. Notably, when motile cells from the 24 h bottom layer are reinoculated into fresh, oxygen-rich medium, BC production resumes, and the medium remains clear in the early stage (image shows 15 h of secondary fermentation), indicating that motile cells can revert to a sessile, BC-producing state (Fig. S8).

### Conclusion

Bacteria use c-di-GMP signaling to achieve precise regulation while minimizing pathway crosstalk, typically through the coordinated action of multiple DGCs and PDEs. In FY-07, three genes encoding c-di-GMP metabolic enzymes are located adjacent to the primary cellulose operon. KoBdcA interacts with KoBdcB and regulates BC production as a DGC via an indirect interaction with BcsA mediated by KoBdcB, whereas KoBpdA functions as a PDE that directly interacts with BcsA. Together, KoBdcA and KoBpdA form an antagonistic DGC-PDE module that maintains intracellular c-di-GMP homeostasis. Their PAS domains mediate DGC-PDE interactions and enable both enzymes to sense oxygen and modulate activity accordingly. During early aerobic fermentation, sufficient oxygen enhances KoBdcA activity, elevating c-di-GMP levels and promoting BC production—a sessile state. As oxygen decreases with fermentation, DGC activity declines, while PDE activity rises, lowering c-di-GMP levels and halting BC synthesis, thus shifting cells to a planktonic, motile state. In summary, KoBdcA and KoBpdA, in coordination with KoBdcB, form an oxygen-responsive regulatory module that balances BC production and motility by tuning intracellular c-di-GMP levels. This study reveals a mechanistic basis for oxygen-mediated c-di-GMP regulation and provides a conceptual framework for understanding similar local signaling systems in other biofilm-forming microorganisms.

## MATERIALS AND METHODS

### Bacterial strains, plasmids, and growth conditions

The bacterial strains and plasmids used in this study are listed in Table S1, and the primers are provided in Table S2 of the supplemental material. *E. coli* s17 (s17) was cultured in Luria-Bertani (LB) medium at 30°C or 37°C and used as the donor strain for conjugative transfer. The BC-producing strain *K. oryzendophytica* FY-07 (FY-07) was used for genetic engineering. During the construction of engineered strains, single colonies of FY-07 transformants were cultured in LB medium.

### Construction, complementation, and overexpression of mutant strains

Gene knockout was performed through homologous recombination, as previously reported (53). The native promoter, together with the full-length gene, was amplified by polymerase chain reaction (PCR) and subsequently cloned into the broad-host-range plasmid pBBR1-MCS2. The recombinant plasmid was then chemically transformed into S17 competent cells. After sequence verification, the transformants were conjugatively transferred into the corresponding knockout and WT strains to generate complementation and overexpression strains, respectively.

### Determination of bacterial growth curves

Single colonies of wild-type or engineered strains were inoculated into 5 mL of LB broth and incubated at 30°C with shaking at 180 rpm for 12 h. Overnight cultures were diluted with fresh LB medium to an OD_600_ of 0.1 and transferred into 100 mL of fresh medium. For anaerobic conditions, all media were boiled under a nitrogen atmosphere to remove dissolved gases, dispensed into anaerobic bottles, and sealed with anaerobic stoppers before incubation at 30°C. For microaerobic conditions, oxygen was removed by nitrogen purging, and cultures were incubated in flasks sealed with standard rubber stoppers (18, 54). Resazurin (1 mg•L^–1^) was used as an indicator to monitor oxygen levels, with the medium retaining its normal color under fully anaerobic conditions and appearing light pink under microaerobic conditions. To prevent interference from BC, 1% (wt/vol) cellulase was added to the medium. Each condition was performed in triplicate. Samples were taken at 1 h intervals, and growth curves were plotted as OD_600_ versus time. Data are presented as mean ± standard deviation.

### Fermentation and yield of BC

Single colonies of FY-07 or engineered strains were streaked onto LB slant agar and incubated at 30°C for 24 h. Bacterial cells were then washed from the slant surface with 100 mL of sterile water. The resulting suspension was used to prepare the fermentation seed culture (≈10⁷ cells•mL⁻¹). For BC fermentation, a 1% (vol/vol) bacterial inoculum was added to fermentation medium containing 2.5 g•L⁻¹ glucose, 7.5 g•L⁻¹ yeast extract, 10 g•L⁻¹ tryptone, 25.2 g•L⁻¹ Na₂HPO₄•12H₂O, 1 g•L⁻¹ KNO₃, and 1 g•L⁻¹ xanthan gum. The culture was statically fermented at 30°C for 24 h, after which the results were observed and photographed. For anaerobic fermentation, the bacterial suspension was inoculated into anaerobic culture bottles and incubated in an anaerobic workstation (DG250, Don Whitley Scientific, UK). L-cysteine hydrochloride (0.5 g•L⁻¹) was added as a reducing agent, and resazurin (1 g•L⁻¹) served as an oxygen indicator. The resulting BC films were washed with distilled water and soaked in 0.1 mol•L⁻¹ NaOH to remove residual cells and medium components, with periodic solution changes until the pellicles turned white. The films were then washed with distilled water until the pH reached 7. The purified BC was freeze-dried, and its dry weight was measured to calculate the yield. For a direct comparison, the BC fermentation results of both single- and double-knockout strains were obtained using the same wild-type (WT) strain as the control.

### Colony morphology assays on Congo red plates

The rdar colony morphology of WT and engineered strains was characterized using Congo Red plates. Bacteria capable of producing exopolysaccharides exhibited distinct rdar colony morphologies on salt-free Congo Red agar plates (55). The salt-free agar medium (10 g•L⁻¹ tryptone, 5 g•L⁻¹ yeast extract, 15 g•L⁻¹ agar) was supplemented with Congo Red (Aladdin, Shanghai, China) at a final concentration of 0.04 g•L⁻¹ and Coomassie Brilliant Blue G-250 (Solarbio, Beijing, China) at 0.02 g•L⁻¹ to enhance color contrast (56). After the bacterial strains were grown to an OD_600_ of 1.0, 3 μL of each suspension was spotted onto the surface of the agar plates and incubated at 30°C for 24 h to observe colony morphology. To avoid potential interference from BC in the experimental measurements, 1% cellulase was incorporated into the bacterial suspension to suppress BC production. For a direct comparison, Congo red plate assay results for both single- and double-knockout strains were obtained using the same WT strain as the control.

### Motility assay

Bacterial swimming and swarming motility were assessed on semi-solid agar plates. The swimming medium contained 1% (wt/vol) tryptone, 0.5% (wt/vol) NaCl, and 0.3% (wt/vol) agar, while the swarming medium consisted of 0.8% (wt/vol) nutrient broth, 0.5% (wt/vol) glucose, and 0.5% (wt/vol) agar (57). To avoid potential interference from BC in the experimental measurements, 1% cellulase was incorporated into the bacterial suspension to suppress BC production. Bacterial cultures were grown to an OD_600_ of 1.0, and 2 μL of each suspension was inoculated at the center of the swarming agar plates. The plates were then incubated at 30°C for 16 h, after which swarming motility was evaluated by photographing the colonies and measuring the diameter of colony expansion. For a direct comparison, motility plate assay results for both single- and double-knockout strains were obtained using the same WT strain as the control.

### *In vitro* enzymatic activity of DGC and PDE

#### *In vitro* PDE enzymatic activity

The FY-07 strain and its respective knockout derivatives carrying the pBTI-*KoBpdA*, pBTI*-KoBpdA*^AAL^ overexpression plasmid, or the pBTI empty vector were cultured overnight at 30°C under induction with 0.5 mM IPTG. The bacterial suspension was adjusted to an OD_600_ of 1.0 (approximately 1 × 10⁹ cells•mL^–1^), then centrifuged at 3,000 × *g* for 15 min. The resulting pellet was resuspended in phosphate-buffered saline (PBS) containing 1 mM phenylmethylsulfonyl fluoride. Cells were disrupted via ultrasonication, and the lysate was centrifuged at 16,100 × *g* for 10 min at 4°C. The supernatant (crude cell extract) was collected for the PDE enzymatic activity assay. PDE activity was evaluated using bis-pNPP as the substrate, following a method modified from a previous report (29). Briefly, the 100 μL reaction mixture contained 50 mM Tris-HCl (pH 7.5), 20 mM MgCl₂, 5 mM bis-pNPP, and 20 μL crude cell extract. The mixture was incubated at 37°C for 60 min, and the release of pNPP was quantified by measuring the absorbance at 410 nm (OD410). A standard curve was constructed using varying concentrations (50–300 µM) of pNPP.

#### *In vitro* DGC enzymatic activity

The FY-07 strain and its respective knockout derivatives carrying the pBTI-*KoBdcA*, pBTI-*KoBdcB*, pBTI-*KoBpdA*, pBTI-*KoBdcA*^GGDEF^, pBTI-*KoBdcB*^GGDEF^ overexpression plasmid, or the pBTI empty vector were cultured, and the supernatant (crude cell extract) was prepared as described in Section 4.6.1. The DGC was assessed by measuring the formation of PPi, a byproduct during the enzymatic conversion of GTP to c-di-GMP. The enzymatic reaction was conducted in a buffer containing 200 mM Tris-HCl (pH 8.0), 100 mM NaCl, 5 mM MgCl2, and 100 μM GTP (58). The reaction mixture was incubated at 30°C for 2 h to facilitate c-di-GMP synthesis. Quantification of PPi was performed using the EnzChek Pyrophosphate Assay Kit (Thermo Fisher Scientific, Cat. #E-6645), following the manufacturer’s protocol. To initiate the detection reaction, 10 µL of inorganic pyrophosphatase (0.03 U) was added to the mixture, followed by incubation at 22°C for 1 h. The absorbance was measured at 360 nm, and background correction was applied by subtracting the absorbance of the control sample lacking PPi. The DGC activity was determined using a standard curve generated with 50–500 μM PPi.

Aerobic conditions were maintained in standard flasks or test tubes. For microaerobic conditions, cultures were incubated in an anaerobic workstation (DG250, Don Whitley Scientific) to ensure a uniform oxygen concentration across all groups, with anaerobic stoppers used to minimize oxygen entry. Anaerobic conditions were established by boiling the medium before sterilization to remove dissolved oxygen, followed by continuous nitrogen flushing until the medium became colorless. In both microaerobic and anaerobic cultures, resazurin (1 mg/L) served as an oxygen indicator, appearing light pink under microaerobic conditions and remaining colorless under anaerobic conditions. For enzymatic assays, cells were cultured under the respective oxygen conditions, harvested, and cell extracts were prepared. Enzymatic activities were then measured either under aerobic conditions or within the anaerobic workstation for microaerobic and anaerobic assays, ensuring consistency between cultivation and measurement environments.

### Bacterial adenylate cyclase two-hybrid (BACTH) assays

The *KoBdcA*, *KoBdcB*, and *KoBpdA* genes, along with their respective individual domains, are cloned from the FY-07 genome into the BACTH vectors pUT18C and pKT25, both encoding N-terminal adenylate cyclase fragment fusions. The ligation products are transformed into *Escherichia coli* DH5α, and positive clones are selected on LB agar plates containing 50 µg/mL^–1^ kanamycin (for pKT25) or 100 µg/mL^–1^ ampicillin (for pUT18C). Positive clones are verified by Sanger sequencing using primers specific to the inserted genes. The correctly sequenced T25 and T18 fusion protein combinations are heat-shocked and transformed into the BACTH-compatible strain BTH101 (Euromedex) for analysis. pUT18C-zip and pKT25-zip serve as positive controls, while the empty pUT18C and pKT25 vectors serve as negative controls. For the assay, 2 µL of overnight-grown BTH101 bacterial suspension containing both plasmids is spotted onto LB agar plates supplemented with 50 µg/mL^–1^ kanamycin, 100 µg/mL^–1^ ampicillin, 0.5 mM IPTG, and 100 µg/mL^–1^ X-Gal (5-bromo-4-chloro-3-indolyl-β-D-galactopyranoside) (59). Plates are incubated at 30°C for 24 h, after which the results are observed and photographed.

### Intracellular c-di-GMP quantification

The extraction and quantification of intracellular c-di-GMP were performed as previously described (60). Bacterial cells were incubated overnight at 30°C in minimal M9 liquid medium, and cultures equivalent to 1 mL at OD600 = 1.8 were collected. The cultures were centrifuged at 16,000 × *g* for 2 min at 4°C, and the supernatants were discarded. The resulting pellets were washed twice with 1 mL of ice-cold PBS to remove residual medium. Each pellet was resuspended in 100 µL of ice-cold PBS and incubated at 100°C for 5 min. Subsequently, ice-cold ethanol was added to a final concentration of 65%, followed by vigorous vortexing for 15 s. The suspension was centrifuged at 16,000 × *g* for 2 min at 4°C, and the supernatant was transferred to a new microcentrifuge tube. This extraction process was repeated thrice, and the combined supernatants were dried using a vacuum freeze dryer (Songyuan Huaxing Technology Co., Ltd., Beijing, China). Prior to analysis, the dried extracts were resuspended in 1 mL of ultrapure water and filtered through a 0.22 µm filter. RP-HPLC analysis was performed using an Agilent ZORBAX StableBond (SB-C18) column (4.6 × 250 mm, 5 µm). The mobile phase consisted of 90% 10 mM ammonium acetate solution and 10% 10 mM ammonium acetate in methanol, with a column temperature of 30°C and a flow rate of 0.2 mL/min. c-di-GMP standards (InvivoGen) at concentrations of 10, 20, 50, 75, and 100 pmol/mL were used to generate a standard curve. The intracellular c-di-GMP concentration was normalized to total cellular protein content (pmol/mg) using the fitted standard curve.

### Protein expression, purification, and UV-Vis analysis of hemin-protein binding

#### Protein expression and purification

The open reading frames encoding the PAS domains of KoBdcA, KoBdcB, and KoBpdA were amplified from FY-07 genomic deoxyribonucleic acid. Point mutants of KoBdcA and KoBpdA were generated using specific primers and cloned into the pET-28a (+) vector via Gibson assembly, yielding recombinant plasmids pET-28a-KoBdcA^PAS^, pET-28a-KoBdcB^PAS^, pET-28a-KoBpdA^PAS^, pET-28a-KoBdcA^PASH19A^, and pET-28a-KoBpdA^PASH21A^. The recombinant plasmids were transformed into *E. coli* BL21(DE3), and positive clones were verified by PCR. Transformed cells were cultured in LB medium containing 50 mg/mL kanamycin at 37°C. When the OD₆₀₀ reached 0.6-0.8, protein expression was induced with 0.5 mM IPTG. Cultures were then incubated at 16°C for 16–20 h, after which cells were harvested and lysed by ultrasonication on ice. Recombinant proteins were purified using Ni-NTA agarose (Smart-Lifesciences, China). Protein purity and molecular weights were analyzed by SDS-PAGE, and protein concentrations were determined using the BCA assay.

#### UV-Vis analysis of hemin-protein binding

The binding of purified PAS proteins to hemin was evaluated using UV-visible spectrophotometry, following previously described methods (45). Hemin (Shanghai YuanYe Biotechnology Co., Ltd., China) was dissolved in TGE buffer (50 mM Tris, 0.5 mM EDTA, 50 mM NaCl, 5% glycerol, pH 7.9) to a final concentration of 12.5 mmol•L⁻¹. The purified PAS proteins were diluted in the same buffer to a final concentration of 12.5 mol•L⁻¹, mixed with the hemin solution at a 1:1 volume ratio, and incubated on ice for 20 min. Spectral scans were recorded over a wavelength range of 250–650 nm using a spectrophotometer, with TGE buffer serving as the baseline blank. Spectra were collected for the PAS protein alone, free hemin, and the PAS-hemin mixture. The resulting absorbance data were exported for subsequent analysis.

### Molecular docking and dynamics simulation

The three-dimensional structures of KoBdcA, KoBdcB, and KoBpdA were retrieved from the AlphaFold Protein Structure Database (61). Molecular docking was performed using AutoDock 4.2 (62). MD simulations of the resulting protein-ligand complexes were conducted with GROMACS v2022.03, employing the AMBER99SB-ILDN force field (63). Each MD simulation was run for 5,000,000 steps, corresponding to a total simulation time of 50 ns. The system was maintained at 300 K and 1 bar using the Berendsen barostat.

### Statistical analysis

Each experimental group was tested in triplicate, and all experiments were independently repeated three times. Data are presented as the mean ± standard deviation to indicate variability around the mean. Statistical analyses were performed using one-way analysis of variance followed by appropriate post-hoc tests for multiple comparisons, or Student’s *t*-test for pairwise comparisons. Differences were considered statistically significant at *P < 0.05, **P < 0.01, and ***P < 0.001, while ns indicates no significance (*P* > 0.05).

## Data Availability

The molecular docking data supporting the findings of this study have been deposited in Zenodo and are publicly available at: https://sandbox.zenodo.org/records/466869. The original data generated and analyzed in this study are included in the article and its supplemental material. Further inquiries can be directed to the corresponding author.

## SUPPLEMENTAL MATERIAL

Supplemental figures and tables spectrum.02292-25-s0001.docx Figures S1 to S8, Tables S1 and S2.

ASM does not own the copyrights to Supplemental Material that may be linked to, or accessed through, an article. The authors have granted ASM a non-exclusive, world-wide license to publish the Supplemental Material files. Please contact the corresponding author directly for reuse.

## References

[1] Hengge R. 2021. High-specificity local and global c-di-GMP signaling. Trends Microbiol 29:993–1003. doi:10.1016/j.tim.2021.02.003 33640237

[2] Valentini M, Filloux A. 2019. Multiple roles of c-di-GMP signaling in bacterial pathogenesis. Annu Rev Microbiol 73:387–406. doi:10.1146/annurev-micro-020518-115555 31500536

[3] Randall TE, Eckartt K, Kakumanu S, Price-Whelan A, Dietrich LEP, Harrison JJ. 2022. Sensory perception in bacterial cyclic diguanylate signal transduction. J Bacteriol 204:e0043321. doi:10.1128/JB.00433-21 34606374 PMC8846402

[4] Zhan X, Zhang K, Wang C, Fan Q, Tang X, Zhang X, Wang K, Fu Y, Liang H. 2024. A c-di-GMP signaling module controls responses to iron in Pseudomonas aeruginosa. Nat Commun 15:1860. doi:10.1038/s41467-024-46149-3 38424057 PMC10904736

[5] Yu Z, Zhang W, Yang H, Chou S-H, Galperin MY, He J. 2023. Gas and light: triggers of c-di-GMP-mediated regulation. FEMS Microbiol Rev 47:fuad034. doi:10.1093/femsre/fuad034 37339911 PMC10505747

[6] Mashruwala AA, Guchte A van de, Boyd JM. 2017. Impaired respiration elicits SrrAB-dependent programmed cell lysis and biofilm formation in Staphylococcus aureus. eLife 6:e23845. doi:10.7554/eLife.23845 28221135 PMC5380435

[7] Walker JA, Rivera S, Weinert EE. 2017. Mechanism and role of globin-coupled sensor signalling. Adv Microb Physiol 71:133–169. doi:10.1016/bs.ampbs.2017.05.003 28760321 PMC6464121

[8] Bouffartigues E, Moscoso JA, Duchesne R, Rosay T, Fito-Boncompte L, Gicquel G, Maillot O, Bénard M, Bazire A, Brenner-Weiss G, Lesouhaitier O, Lerouge P, Dufour A, Orange N, Feuilloley MGJ, Overhage J, Filloux A, Chevalier S. 2015. The absence of the Pseudomonas aeruginosa OprF protein leads to increased biofilm formation through variation in c-di-GMP level. Front Microbiol 6:630. doi:10.3389/fmicb.2015.00630 26157434 PMC4477172

[9] Boehm A, Steiner S, Zaehringer F, Casanova A, Hamburger F, Ritz D, Keck W, Ackermann M, Schirmer T, Jenal U. 2009. Second messenger signalling governs Escherichia coli biofilm induction upon ribosomal stress. Mol Microbiol 72:1500–1516. doi:10.1111/j.1365-2958.2009.06739.x 19460094

[10] Pérez-Mendoza D, Rodríguez-Carvajal MÁ, Romero-Jiménez L, Farias G de A, Lloret J, Gallegos MT, Sanjuán J. 2015. Novel mixed-linkage β-glucan activated by c-di-GMP in Sinorhizobium meliloti. Proc Natl Acad Sci USA 112:E757–E765. doi:10.1073/pnas.1421748112 25650430 PMC4343143

[11] Conner JG, Zamorano-Sánchez D, Park JH, Sondermann H, Yildiz FH. 2017. The ins and outs of cyclic di-GMP signaling in Vibrio cholerae. Curr Opin Microbiol 36:20–29. doi:10.1016/j.mib.2017.01.002 28171809 PMC5534393

[12] Junkermeier EH, Hengge R. 2023. Local signaling enhances output specificity of bacterial c-di-GMP signaling networks. microLife 4:uqad026. doi:10.1093/femsml/uqad026 37251514 PMC10211494

[13] Van Loon JC, Whitfield GB, Wong N, O’Neal L, Henrickson A, Demeler B, O’Toole GA, Parsek MR, Howell PL. 2024. Binding of GTP to BifA is required for the production of Pel-dependent biofilms in Pseudomonas aeruginosa. J Bacteriol 206:e0033123. doi:10.1128/jb.00331-23 38197635 PMC10882990

[14] Dahlstrom KM, Giglio KM, Collins AJ, Sondermann H, O’Toole GA. 2015. Contribution of physical interactions to signaling specificity between a diguanylate cyclase and its effector. mBio 6:e01978-15. doi:10.1128/mBio.01978-15 26670387 PMC4676286

[15] Sarenko O, Klauck G, Wilke FM, Pfiffer V, Richter AM, Herbst S, Kaever V, Hengge R. 2017. More than enzymes that make or break cyclic di-GMP—local signaling in the interactome of GGDEF/EAL domain proteins of Escherichia coli. mBio 8:e01639-17. doi:10.1128/mBio.01639-17 29018125 PMC5635695

[16] Ross P, Weinhouse H, Aloni Y, Michaeli D, Weinberger-Ohana P, Mayer R, Braun S, de Vroom E, van der Marel GA, van Boom JH, Benziman M. 1987. Regulation of cellulose synthesis in Acetobacter xylinum by cyclic diguanylic acid. Nature 325:279–281. doi:10.1038/325279a0 18990795

[17] Gao Ge, Zhang Y, Niu S, Chen Y, Wang S, Anwar N, Chen S, Li G, Ma T. 2022. Reclassification of Enterobacter sp. FY-07 as Kosakonia oryzendophytica FY-07 and its potential to promote plant growth. Microorganisms 10:575. doi:10.3390/microorganisms10030575 35336150 PMC8951479

[18] Gao G., Liao Z, Cao Y, Zhang Y, Zhang Y, Wu M, Li G, Ma T. 2021. Highly efficient production of bacterial cellulose from corn stover total hydrolysate by Enterobacter sp. FY-07. Bioresour Technol 341:125781. doi:10.1016/j.biortech.2021.125781 34454235

[19] Römling U, Gomelsky M, Galperin MY. 2005. C-di-GMP: the dawning of a novel bacterial signalling system. Mol Microbiol 57:629–639. doi:10.1111/j.1365-2958.2005.04697.x 16045609

[20] Sayers EW, Beck J, Brister JR, Bolton EE, Canese K, Comeau DC, Funk K, Ketter A, Kim S, Kimchi A, Kitts PA, Kuznetsov A, Lathrop S, Lu Z, McGarvey K, Madden TL, Murphy TD, O’Leary N, Phan L, Schneider VA, Thibaud-Nissen F, Trawick BW, Pruitt KD, Ostell J. 2020. Database resources of the National Center for Biotechnology Information. Nucleic Acids Res 48:D9–D16. doi:10.1093/nar/gkz899 31602479 PMC6943063

[21] Ji K, Wang W, Zeng B, Chen S, Zhao Q, Chen Y, Li G, Ma T. 2016. Bacterial cellulose synthesis mechanism of facultative anaerobe Enterobacter sp. FY-07. Sci Rep 6:21863. doi:10.1038/srep21863 26911736 PMC4766428

[22] Bateman A, Martin M-J, Orchard S, Magrane M, Adesina A, Ahmad S, Bowler-Barnett EH, Bye-A-Jee H, Carpentier D, Denny P, et al.. 2025. UniProt: the Universal Protein Knowledgebase in 2025. Nucleic Acids Res 53:D609–D617. doi:10.1093/nar/gkae1010 39552041 PMC11701636

[23] Chen Y, Liu S, Liu C, Huang Y, Chi K, Su T, Zhu D, Peng J, Xia Z, He J, Xu S, Hu W, Gu L. 2016. Dcsbis (PA2771) from Pseudomonas aeruginosa is a highly active diguanylate cyclase with unique activity regulation. Sci Rep 6:29499. doi:10.1038/srep29499 27388857 PMC4937426

[24] Yao Y, Xi N, Hai E, Zhang X, Guo J, Lin Z, Huang W. 2023. PA0575 (RmcA) interacts with other c-di-GMP metabolizing proteins in Pseudomonas aeruginosa PAO1. J Gen Appl Microbiol 68:232–241. doi:10.2323/jgam.2022.05.003 35732459

[25] Severin GB, Waters CM. 2017. Spectrophotometric and mass spectroscopic methods for the quantification and kinetic evaluation of in vitro c-di-GMP synthesis, p 71–84. *In* Methods in Molecular Biology. Vol. 1657. Humana Press, New York. 28889287 10.1007/978-1-4939-7240-1_7

[26] Schirmer T. 2016. C-di-GMP synthesis: structural aspects of evolution, catalysis and regulation. J Mol Biol 428:3683–3701. doi:10.1016/j.jmb.2016.07.023 27498163

[27] Schirmer T, Jenal U. 2009. Structural and mechanistic determinants of c-di-GMP signalling. Nat Rev Microbiol 7:724–735. doi:10.1038/nrmicro2203 19756011

[28] Chan C, Paul R, Samoray D, Amiot NC, Giese B, Jenal U, Schirmer T. 2004. Structural basis of activity and allosteric control of diguanylate cyclase. Proc Natl Acad Sci USA 101:17084–17089. doi:10.1073/pnas.0406134101 15569936 PMC535365

[29] Liu X, Beyhan S, Lim B, Linington RG, Yildiz FH. 2010. Identification and characterization of a phosphodiesterase that inversely regulates motility and biofilm formation in Vibrio cholerae. J Bacteriol 192:4541–4552. doi:10.1128/JB.00209-10 20622061 PMC2937418

[30] Cimdins A, Simm R. 2017. Semiquantitative analysis of the red, dry, and rough colony morphology of *Salmonella enterica* serovar Typhimurium and *Escherichia coli* using Congo red, p 225–241. *In* Sauer K (ed), C-di-GMP signaling: methods and protocols. Springer New York, New York, NY. 10.1007/978-1-4939-7240-1_18 28889298

[31] Kirisits MJ, Prost L, Starkey M, Parsek MR. 2005. Characterization of colony morphology variants isolated from Pseudomonas aeruginosa biofilms. Appl Environ Microbiol 71:4809–4821. doi:10.1128/AEM.71.8.4809-4821.2005 16085879 PMC1183349

[32] Morgan JLW, McNamara JT, Zimmer J. 2014. Mechanism of activation of bacterial cellulose synthase by cyclic di-GMP. Nat Struct Mol Biol 21:489–496. doi:10.1038/nsmb.2803 24704788 PMC4013215

[33] Guan C, Huang Y, Zhou Y, Han Y, Liu S, Liu S, Kong W, Wang T, Zhang Y. 2024. FlhF affects the subcellular clustering of WspR through HsbR in Pseudomonas aeruginosa. Appl Environ Microbiol 90. doi:10.1128/aem.01548-23 PMC10807432 38112425

[34] Tuckerman JR, Gonzalez G, Sousa EHS, Wan X, Saito JA, Alam M, Gilles-Gonzalez M-A. 2009. An oxygen-sensing diguanylate cyclase and phosphodiesterase couple for c-di-GMP control. Biochemistry 48:9764–9774. doi:10.1021/bi901409g 19764732

[35] Fu Y, Yu Z, Liu S, Chen B, Zhu L, Li Z, Chou S-H, He J. 2018. c-di-GMP regulates various phenotypes and insecticidal activity of Gram-positive Bacillus thuringiensis. Front Microbiol 9:2018. doi:10.3389/fmicb.2018.00045 29487570 PMC5816809

[36] Jenal U, Reinders A, Lori C. 2017. Cyclic di-GMP: second messenger extraordinaire. Nat Rev Microbiol 15:271–284. doi:10.1038/nrmicro.2016.190 28163311

[37] Kearns DB. 2010. A field guide to bacterial swarming motility. Nat Rev Microbiol 8:634–644. doi:10.1038/nrmicro2405 20694026 PMC3135019

[38] Fitzgerald DM, Bonocora RP, Wade JT. 2014. Comprehensive mapping of the Escherichia coli flagellar regulatory network. PLoS Genet 10:e1004649. doi:10.1371/journal.pgen.1004649 25275371 PMC4183435

[39] Shahid M, Al-Khattaf FS, Danish M, Zeyad MT, Atef Hatamleh A, Mohamed A, Ali S. 2022. PGPR Kosakonia radicincitans KR-17 increases the salt tolerance of radish by regulating ion-homeostasis, photosynthetic molecules, redox potential, and stressor metabolites. Front Plant Sci 13:919696. doi:10.3389/fpls.2022.919696 35979076 PMC9376370

[40] Patrick JE, Kearns DB. 2012. Swarming motility and the control of master regulators of flagellar biosynthesis. Mol Microbiol 83:14–23. doi:10.1111/j.1365-2958.2011.07917.x 22092493 PMC3245337

[41] Henry JT, Crosson S. 2011. Ligand-binding PAS domains in a genomic, cellular, and structural context. Annu Rev Microbiol 65:261–286. doi:10.1146/annurev-micro-121809-151631 21663441 PMC3298442

[42] Van Loon JC, Whitfield GB, Wong N, O’Neal L, Henrickson A, Demeler B, O’Toole GA, Parsek MR, Howell PL. 2024. Binding of GTP to BifA is required for the production of Pel-dependent biofilms in Pseudomonas aeruginosa. J Bacteriol 206. doi:10.1128/jb.00331-23 PMC10882990 38197635

[43] Stuffle EC, Johnson MS, Watts KJ. 2021. PAS domains in bacterial signal transduction. Curr Opin Microbiol 61:8–15. doi:10.1016/j.mib.2021.01.004 33647528 PMC8169565

[44] Richter AM, Possling A, Malysheva N, Yousef KP, Herbst S, von Kleist M, Hengge R. 2020. Local c-di-GMP signaling in the control of synthesis of the E. coli biofilm exopolysaccharide pEtN-cellulose. J Mol Biol 432:4576–4595. doi:10.1016/j.jmb.2020.06.006 32534064 PMC7397504

[45] Sawai H, Yoshioka S, Uchida T, Hyodo M, Hayakawa Y, Ishimori K, Aono S. 2010. Molecular oxygen regulates the enzymatic activity of a heme-containing diguanylate cyclase (HemDGC) for the synthesis of cyclic di-GMP. Biochim Biophys Acta 1804:166–172. doi:10.1016/j.bbapap.2009.09.028 19818878

[46] Das D, Patra M, Chakrabarti A. 2015. Binding of hemin, hematoporphyrin, and protoporphyrin with erythroid spectrin: fluorescence and molecular docking studies. Eur Biophys J 44:171–182. doi:10.1007/s00249-015-1012-2 25737232

[47] Wu W, Kumar P, Brautigam CA, Tso S-C, Baniasadi HR, Kober DL, Gilles-Gonzalez M-A. 2024. Structures of the multi-domain oxygen sensor DosP: remote control of a c-di-GMP phosphodiesterase by a regulatory PAS domain. Nat Commun 15:9653. doi:10.1038/s41467-024-53942-7 39511182 PMC11543664

[48] Tarnawski M, Barends TRM, Schlichting I. 2015. Structural analysis of an oxygen-regulated diguanylate cyclase. Acta Crystallogr D Biol Crystallogr 71:2158–2177. doi:10.1107/S139900471501545X 26527135

[49] Sarkar J, Miller DP, Oliver LD, Marconi RT. 2018. The Treponema denticola PAS domain-containing histidine kinase Hpk2 is a heme binding sensor of oxygen levels. J Bacteriol 200:e00116-18. doi:10.1128/JB.00116-18 29986942 PMC6112003

[50] Lechuga GC, Napoleão-Pêgo P, Morel CM, Provance DW, De-Simone SG. 2022. New insights into hemopexin-binding to hemin and hemoglobin. Int J Mol Sci 23:3789. doi:10.3390/ijms23073789 35409149 PMC8998376

[51] Schuelke-Sanchez A, Yennawar NH, Weinert EE. 2024. Oxygen-selective regulation of cyclic di-GMP synthesis by a globin coupled sensor with a shortened linking domain modulates Shewanella sp. ANA-3 biofilm. J Inorg Biochem 252:112482. doi:10.1016/j.jinorgbio.2024.112482 38218138 PMC11616453

[52] Vávra J, Sergunin A, Jeřábek P, Shimizu T, Martínková M. 2022. Signal transduction mechanisms in heme-based globin-coupled oxygen sensors with a focus on a histidine kinase (AfGcHK) and a diguanylate cyclase (YddV or EcDosC). Biol Chem 403:1031–1042. doi:10.1515/hsz-2022-0185 36165459

[53] Gao G, Ji K, Zhang Y, Liu X, Dai X, Zhi B, Cao Y, Liu D, Wu M, Li G, Ma T. 2020. Microbial enhanced oil recovery through deep profile control using a conditional bacterial cellulose-producing strain derived from Enterobacter sp. FY-07. Microb Cell Fact 19:59. doi:10.1186/s12934-020-01314-3 32138785 PMC7059367

[54] Ma T, Ji K, Wang W, Wang J, Li Z, Ran H, Liu B, Li G. 2012. Cellulose synthesized by Enterobacter sp. FY-07 under aerobic and anaerobic conditions. Bioresour Technol 126:18–23. doi:10.1016/j.biortech.2012.09.040 23073085

[55] Liu Y, Ma Y, Ma Z, Han X, Qi H, Andersen JB, Xu H, Tolker-Nielsen T, Qiao M. 2021. Redox protein OsaR (PA0056) regulates dsbM and the oxidative stress response in Pseudomonas aeruginosa. Antimicrob Agents Chemother 65. doi:10.1128/AAC.01771-20 PMC8092530 33361299

[56] Jain S, Chen J. 2006. Antibiotic resistance profiles and cell surface components of Salmonellae. J Food Prot 69:1017–1023. doi:10.4315/0362-028x-69.5.1017 16715798

[57] Inoue T, Shingaki R, Fukui K. 2008. Inhibition of swarming motility of Pseudomonas aeruginosa by branched-chain fatty acids. FEMS Microbiol Lett 281:81–86. doi:10.1111/j.1574-6968.2008.01089.x 18318842

[58] Oliveira MC, Teixeira RD, Andrade MO, Pinheiro GMS, Ramos CHI, Farah CS. 2015. Cooperative substrate binding by a diguanylate cyclase. J Mol Biol 427:415–432. doi:10.1016/j.jmb.2014.11.012 25463434

[59] Zhang T, Zhang S, Wang Y, Peng Z, Xin B, Zhong C. 2025. Tandem GGDEF–EAL domain proteins pleiotropically modulate c-di-GMP metabolism enrolled in bacterial cellulose biosynthesis. J Agric Food Chem 73:1982–1993. doi:10.1021/acs.jafc.4c07301 39794331

[60] Petrova OE, Sauer K. 2017. High-performance liquid chromatography (HPLC)-based detection and quantitation of cellular c-di-GMP, p 33–43. *In* Sauer K (ed), C-di-GMP signaling: methods and protocols. Springer New York, New York, NY. 10.1007/978-1-4939-7240-1_4 PMC5702474 28889284

[61] Varadi M, Anyango S, Deshpande M, Nair S, Natassia C, Yordanova G, Yuan D, Stroe O, Wood G, Laydon A, et al.. 2022. AlphaFold protein structure database: massively expanding the structural coverage of protein-sequence space with high-accuracy models. Nucleic Acids Res 50:D439–D444. doi:10.1093/nar/gkab1061 34791371 PMC8728224

[62] Morris GM, Huey R, Lindstrom W, Sanner MF, Belew RK, Goodsell DS, Olson AJ. 2009. AutoDock4 and AutoDockTools4: automated docking with selective receptor flexibility. J Comput Chem 30:2785–2791. doi:10.1002/jcc.21256 19399780 PMC2760638

[63] Abraham MJ, Murtola T, Schulz R, Páll S, Smith JC, Hess B, Lindahl E. 2015. GROMACS: high performance molecular simulations through multi-level parallelism from laptops to supercomputers. SoftwareX 1–2:19–25. doi:10.1016/j.softx.2015.06.001

