# Supplementary material for: Oxygen-responsive KoBdcA/KoBdcB/KoBpdA system regulates c-di-GMP levels to control bacterial cellulose biosynthesis and motility in Kosakonia oryzendophytica FY-07
Source: Microbiol Spectr. 2026 Mar 26;14(5):e02292-25. doi: 10.1128/spectrum.02292-25 (PMC13141831; doi:10.1128/spectrum.02292-25)
Supplement: Supplemental figures and tables — Figures S1 to S8, Tables S1 and S2. [file spectrum.02292-25-s0001.docx]

Supporting Information

Oxygen-responsive KoBdcA/KoBdcB/KoBpdA system regulates c-di-GMP levels to control bacterial cellulose biosynthesis and motility in *Kosakonia oryzendophytica* FY-07

Xueqing Zhao^a^, Yucheng Shi^a^, Wenzhuo Tian^a^, Yutong Tian^a^, Chuxiao Hu^a^, Ziye Guo^a^, Ting Ma^a,b,c^*, Guoqiang Li^a,b^*, Ge Gao^a,d^*

^a^ *College of Life Sciences, Nankai University, Tianjin, 300071, China*

^b^ *Key Laboratory of Molecular Microbiology and Technology, Ministry of Education**, Nankai University, Tianjin, 300071, China*

^c^ *Tianjin Engineering Technology Center of Green Manufacturing Biobased Materials, Tianjin, 300071, China*

^d^ *School of Life Science, Shanxi University, Taiyuan, 030006, China*

*Corresponding authors

Ting Ma: [tingma@nankai.edu.cn](mailto:tingma@nankai.edu.cn.)

Guoqiang Li: [gqli](mailto:gqli@nankai.edu.cn)[@nankai.edu.cn](mailto:gqli@nankai.edu.cn)

Ge Gao: gaoge@mail.nankai.edu.cn


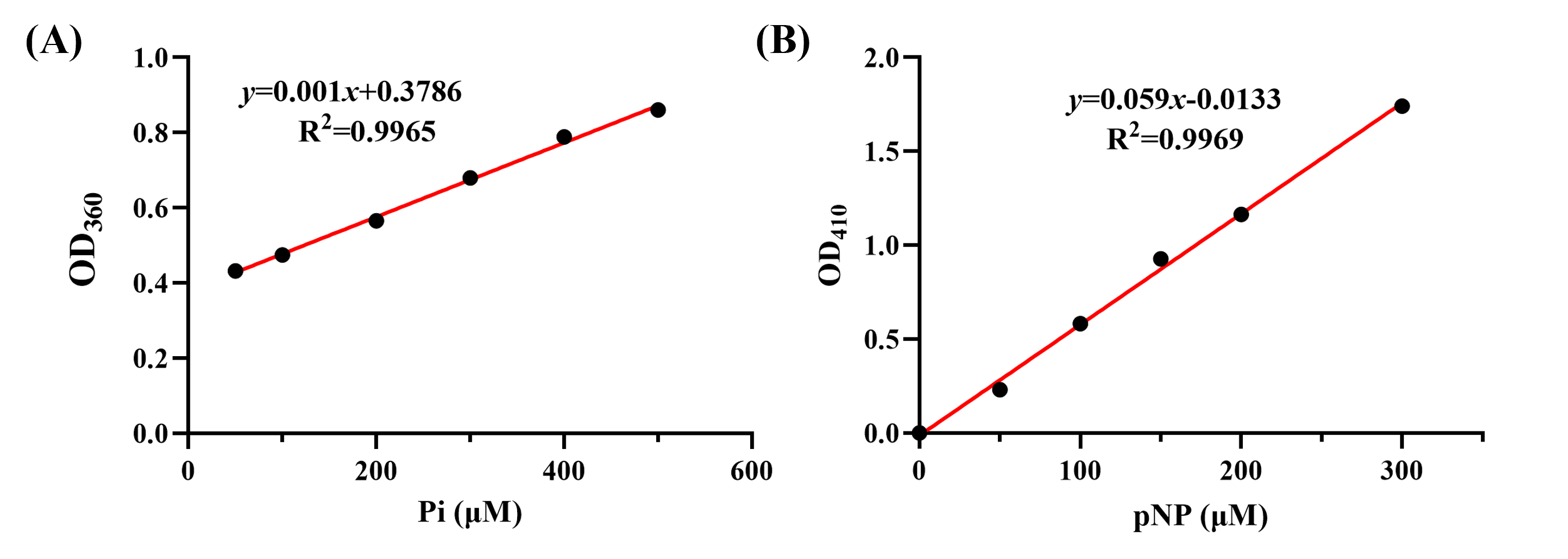


Fig. S1: Standard curves of Pi and pNP.


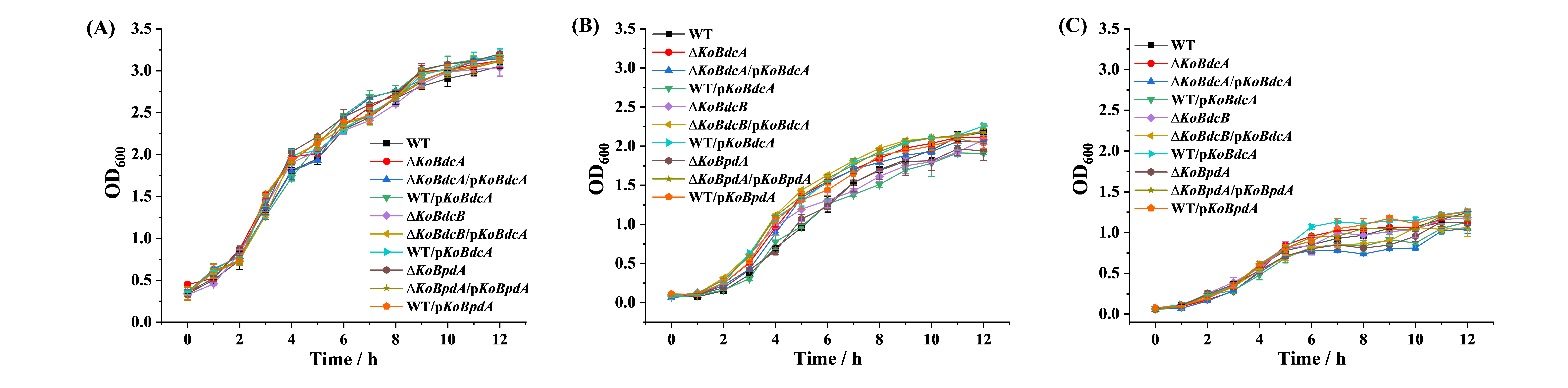


Fig. S2. Growth curves of FY-07 WT, *∆KoBdcA*, *∆KoBdcB*, *∆KoBpdA*, and their complementation and overexpression strains under (A) aerobic, (B) microaerobic, and (C) anaerobic conditions.


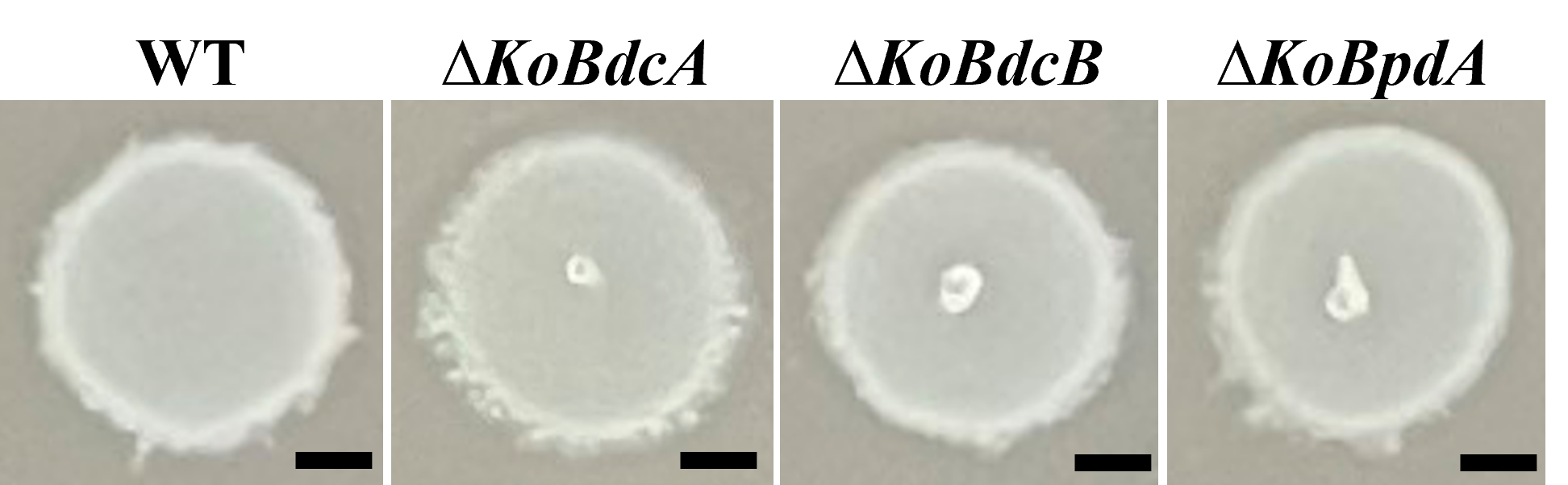


Fig. S3: Swimming motility of FY-07 WT, *∆KoBdcA*, *∆KoBdcB*, and *∆KoBpdA* strains. Scale bar: 0.2 cm.


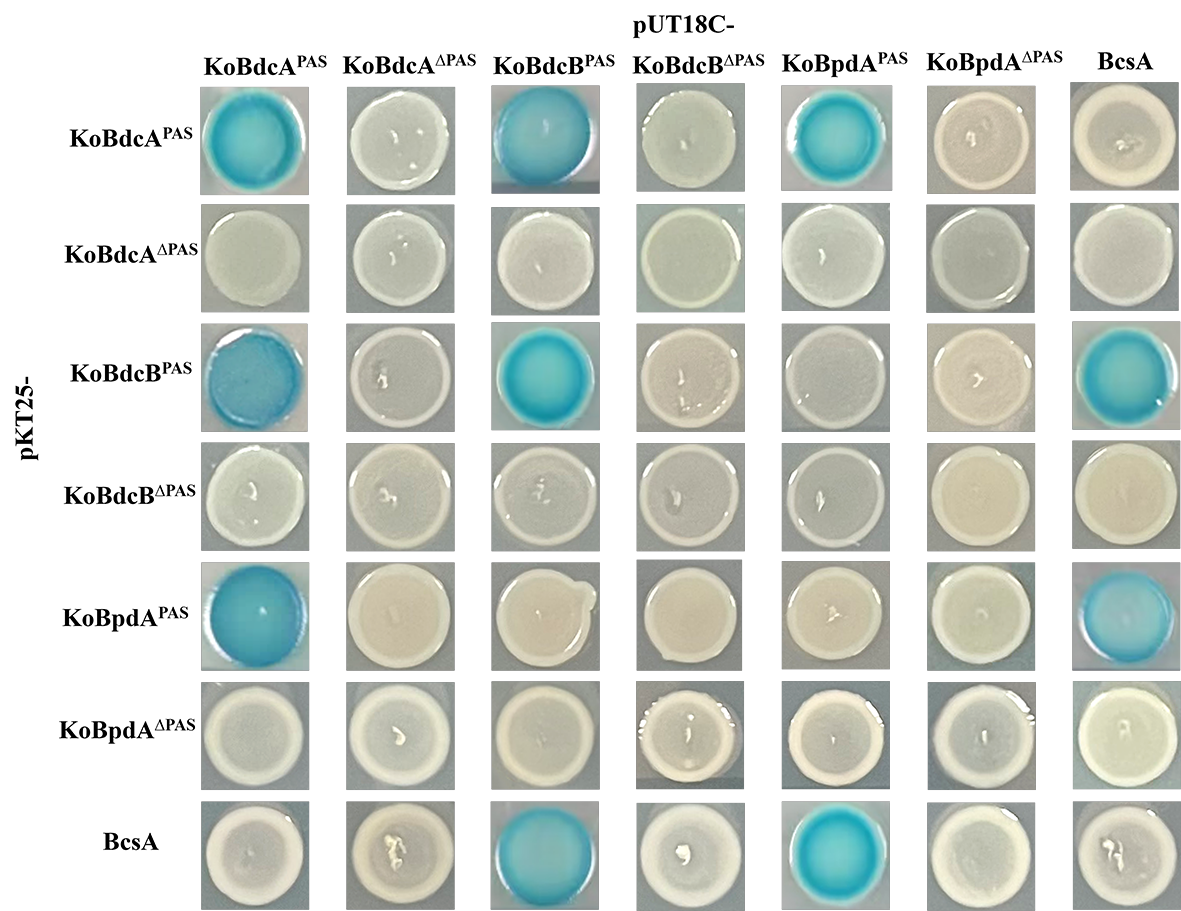


Fig. S4: Interactions between the PAS domains of KoBdcA, KoBdcB, and KoBpdA, or their PAS domain deletion mutants, and the cellulose synthase subunit BcsA were analyzed using a bacterial two-hybrid system.


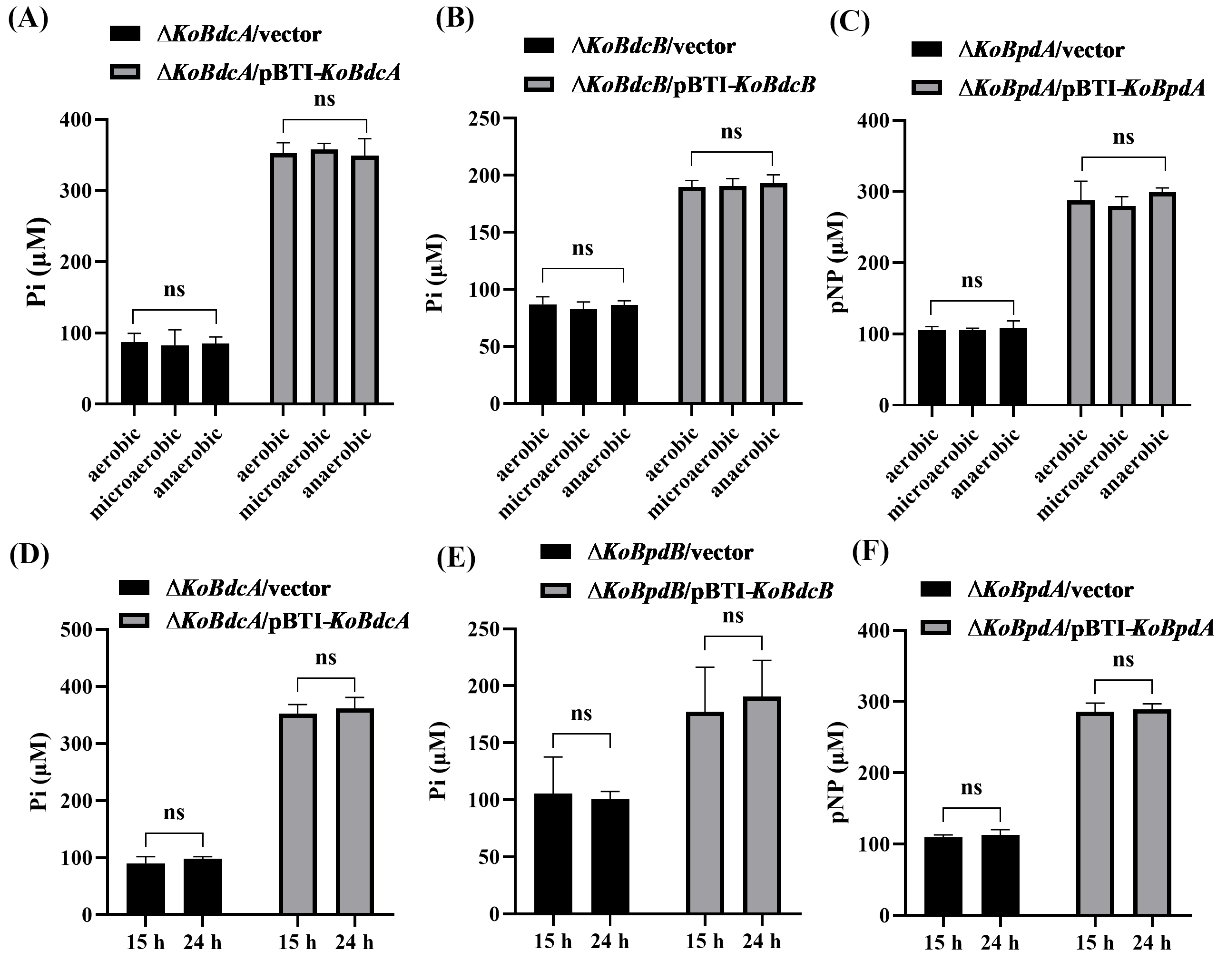


Fig. S5: (A-C) Enzymatic activities of KoBdcA, KoBdcB, and KoBpdA as DGCs or PDEs in their respective knockout strains under aerobic, microaerobic, and anaerobic conditions. (D-F) Comparison of DGC or PDE activities of KoBdcA, KoBdcB, and KoBpdA in knockout strains at 15 h and 24 h of fermentation.


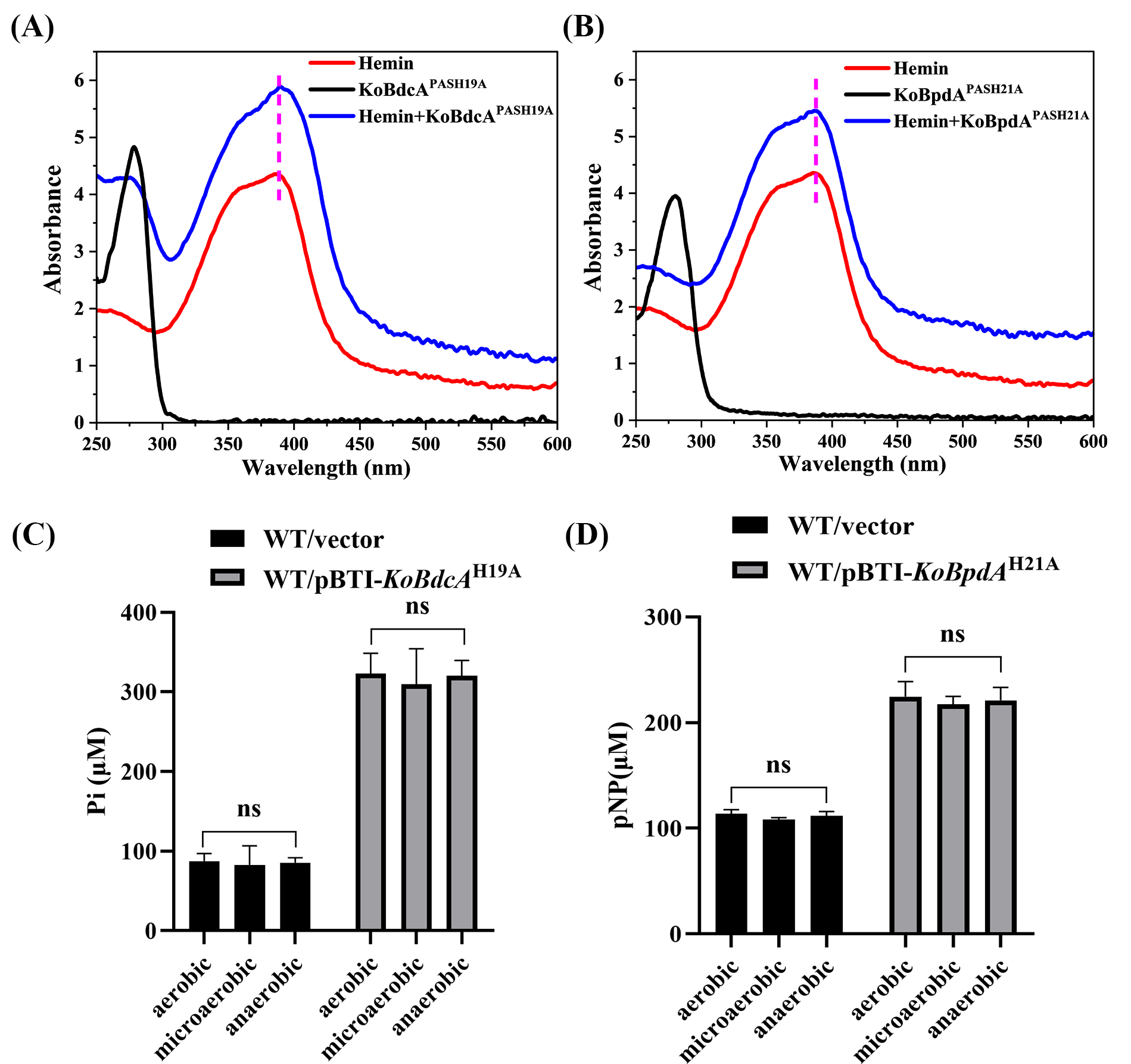


Fig. S6: UV-Vis absorption spectra of (A) KoBdcA and (B) KoBpdA PAS domain mutant proteins after incubation with hemin. Enzymatic activities of (C) KoBdcA and (D) KoBpdA histidine mutants as DGCs and PDEs under aerobic, microaerobic, and anaerobic conditions.


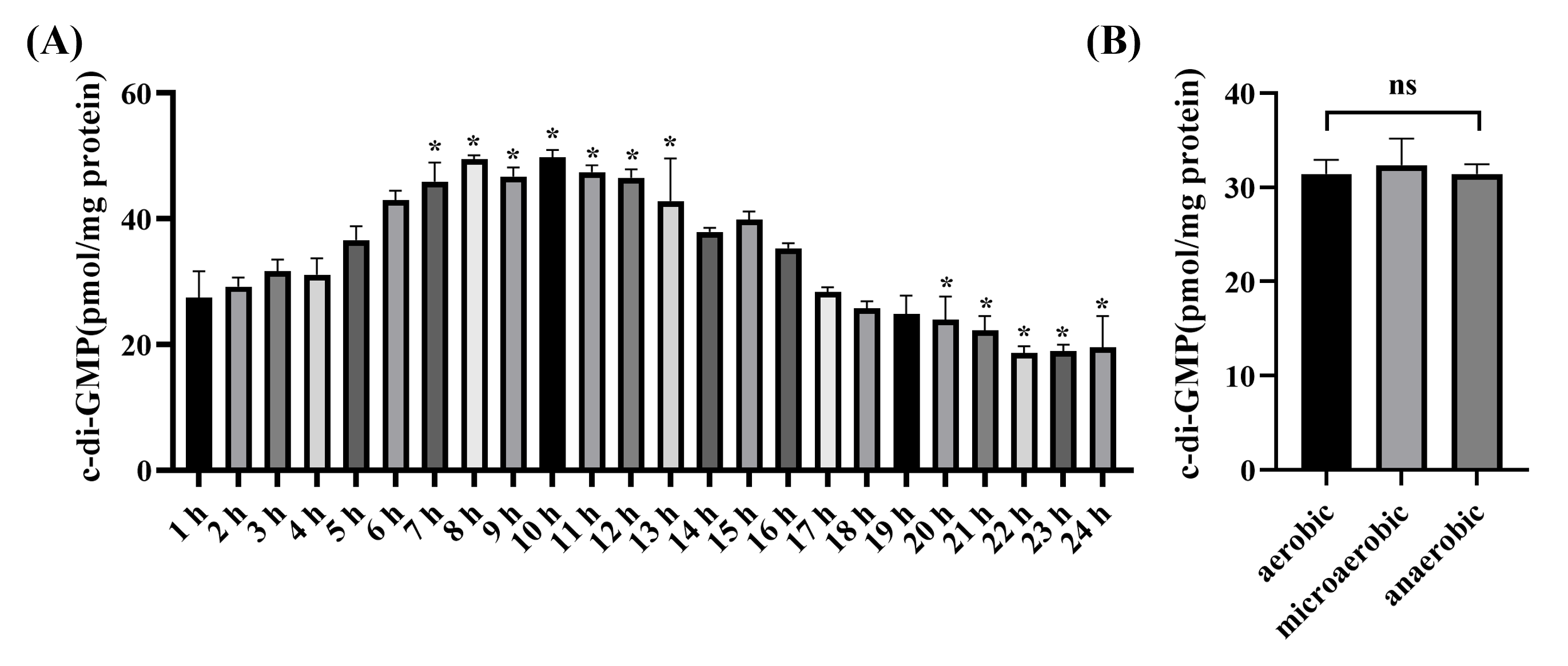


Fig. S7: (A) Hourly measurements of intracellular c-di-GMP levels in FY-07 WT during aerobic fermentation. (B) Intracellular c-di-GMP levels in FY-07 WT during the logarithmic growth phase under aerobic, microaerobic, and anaerobic conditions.


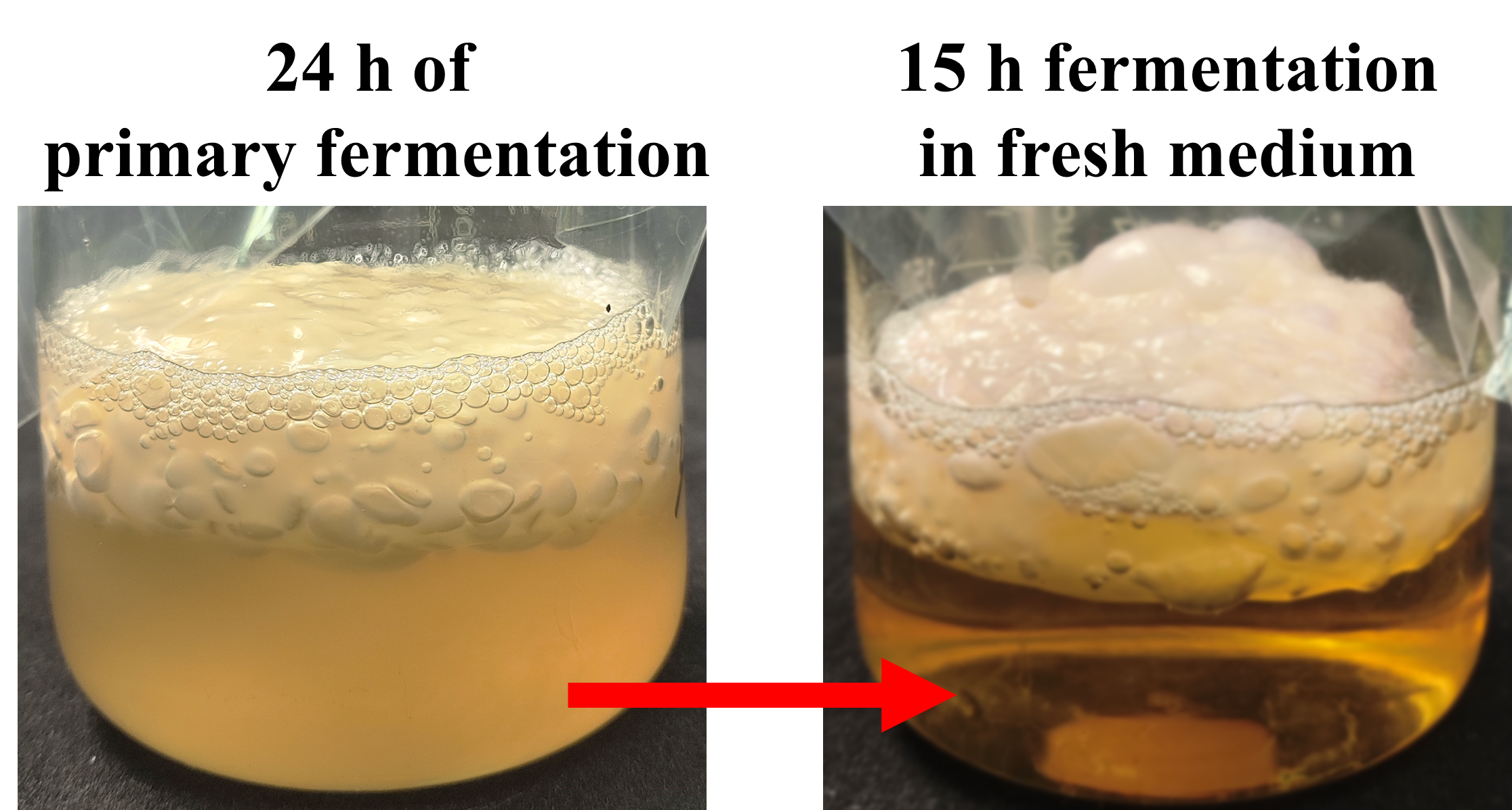


Fig. S8: Secondary fermentation (15 h) initiated by inoculating bottom-layer cells from 24 h primary fermentation into fresh medium.

Table S1. Bacterial strains and plasmids used in this study

| Bacterial strains or plasmids | Relevant characteristics | Sources or references |
| --- | --- | --- |
| Bacterial strains |  |  |
| *E. coli* |  |  |
| s17 | *rec*A pro *hsd*R RP4-2-Tc::Mu-Km::Tn7 | Lab collection |
| DH5α | F^−^ϕ80 lacZΔM15Δ(*lac*ZYA-*arg*F)U169 *end* A1 *rec* A1 *hsd*R17(rk^−^, mk^−^)  *sup* E44 λ^−^ thi-1 *gyr*A96 *rel*A1 *pho*A | Lab collection |
| BL21(DE3) | Expression strain; F^−^ *omp*T *hsdSB* (rB^−^ , mB^−^ ) *gal dcm* (DE3) | Lab collection |
| BL21(DE3)/pET28a-KoBdcA^PAS^ | BL21(DE3) harboring pET28a-KoBdcA^PAS^ plasmid | This study |
| BL21(DE3)/pET28a-KoBdcB^PAS^ | BL21(DE3) harboring pET28a-KoBdcB^PAS^ plasmid | This study |
| BL21(DE3)/pET28a-KoBpdA^PAS^ | BL21(DE3) harboring pET28a-KoBpdA^PAS^ plasmid | This study |
| BTH101 | F^−^, *cya*^−^99, *araD*139, *galE*15, *galK*16, *rpsL1* (*Str^r^*), *hsdR2*, *mcrA1*, *mcrB1* | Lab collection |
| *Kosakonia oryzendophytica* |  |  |
| FY-07 | wild-type strain | This lab |
| WT/vector | WT containing pBTI plasmid | This study |
| WT/vector-KoBdcA | WT containing pBTI-KoBdcA plasmid | This study |
| WT/vector-KoBdcB | WT containing pBTI-KoBdcB plasmid | This study |
| WT/vector-KoBpdA | WT containing pBTI-KoBpdA plasmid | This study |
| WT/vector-KoBdcA^GGDEF^ | WT containing pBTI-KoBdcA^GGDEF^ plasmid | This study |
| WT/vector-KoBdcB^GGDEF^ | WT containing pBTI-KoBdcB^GGDEF^ plasmid | This study |
| WT/vector-KoBpdA^AAL^ | WT containing pBTI-KoBpdA^AAL^ plasmid | This study |
| *∆**KoBdcA* | *KoBdcA* gene deletion mutant | This study |
| *∆KoBdcA*/p*KoBdcA* | *KoBdcA* complemented strain (*∆KoBdcA* harboring pBBR-KoBdcA vector) | This study |
| WT/p*KoBdcA* | *KoBdcA* overexpressing strain (WT harboring pBBR-KoBdcA vector) | This study |
| *∆KoBdcB* | *KoBdcB* gene deletion mutant | This study |
| *∆KoBdcB*/p*KoBdcB* | *KoBdcB* complemented strain (*∆KoBdcB* harboring pBBR-KoBdcB vector) | This study |
| WT/p*KoBdcB* | *KoBdcB* overexpressing strain (WT harboring pBBR-KoBdcB vector) | This study |
| *∆**KoBpdA* | *KoBpdA* gene deletion mutant | This study |
| *∆KoBpdA*/p*KoBpdA* | *KoBpdA* complemented strain (*∆KoBpdA* harboring pBBR-KoBpdA vector) | This study |
| WT/p*KoBpdA* | *KoBpdA* overexpressing strain (WT harboring pBBR-KoBpdA vector) | This study |
| ∆*KoBdcA* ∆*KoBdcB* | *KoBdcA* *KoBdcB* double-knockout gene deletion mutant | This study |
| ∆*KoBdcA* ∆*KoBdcB*/p*KoBdcB* | *KoBdcB* complemented strain (∆*KoBdcA* ∆*KoBdcB* harboring pBBR-KoBdcB vector) | This study |
| ∆*KoBdcA* ∆*KoBdcB*/p*KoBdcA* | *KoBdcA* complemented strain (∆*KoBdcA* ∆*KoBdcB* harboring pBBR-KoBdcA vector) | This study |
| ∆*KoBdcA* ∆*KoBpdA* | *KoBdcA* *KoBpdA* double-knockout gene deletion mutant | This study |
| ∆*KoBdcA* ∆*KoBpdA*/p*KoBdcA* | *KoBdcA* complemented strain (∆*KoBdcA* ∆*KoBdcA* harboring pBBR-KoBdcA vector) | This study |
| ∆*KoBdcA* ∆*KoBpdA*/p*KoBpdA* | *KoBpdA* complemented strain (∆*KoBdcA* ∆*KoBpdA* harboring pBBR-KoBpdA vector) | This study |
| ∆*KoBdcB* ∆*KoBpdA* | *KoBdcB* *KoBpdA* double-knockout gene deletion mutant | This study |
| ∆*KoBdcB* ∆*KoBpdA*/p*KoBdcB* | *KoBdcB* complemented strain (∆*KoBdcA* ∆*KoBpdA* harboring pBBR-KoBpdB vector) | This study |
| *∆KoBdcB ∆KoBpdA/pKoBpdA* | *KoBdcB* complemented strain (∆*KoBdcB* ∆*KoBpdA* harboring pBBR-KoBpdA vector) | This study |
| Plasmids |  |  |
| pTSK2 | Ts; *sac*B; Cm ^r^; Tc ^r^ ; oriT | This lab |
| pBBR1MCS-2 | pBBR1 oriV; pBBR1 Rep; Kan^r^; oriT | [1] |
| pBBR-KoBdcA | pBBR1MCS-2 derivative expressing *KoBdcA* | This study |
| pBBR-KoBdcB | pBBR1MCS-2 derivative expressing *KoBdcB* | This study |
| pBBR-KoBpdA | pBBR1MCS-2 derivative expressing *KoBpdA* | This study |
| pBTI | A vector from pBBR1MCS-2, pBBR1 oriV; P_tac_, kan^r^, lacI | [2] |
| pBTI-KoBdcA | pBTI containing the coding sequence of KoBdcA, Km^r^ | This study |
| pBTI-KoBdcB | pBTI containing the coding sequence of KoBdcB, Km^r^ | This study |
| pBTI-KoBpdA | pBTI containing the coding sequence of KoBpdA, Km^r^ | This study |
| pBTI-KoBdcA^GGDEF^ | pBTI containing the coding sequence of GGDEF domain of KoBpdA, Km^r^ | This study |
| pBTI-KoBdcB^GGDEF^ | pBTI containing the coding sequence of GGDEF domain of KoBdcB, Km^r^ | This study |
| pBTI-KoBpdA^AAL^ | pBTI containing the coding sequence of KoBpdA with a E36A mutation in the EAL domain, Km^r^ | This study |
| pET-28a (+) | Expression vector, Kan^r^ | Lab collection |
| pET-28a (+)-KoBdcA^PAS^ | pET-28a (+) harboring the coding sequence of PAS domain of KoBdcA, Km^r^ | This study |
| pET-28a (+)-KoBdcB^PAS^ | pET-28a (+) harboring the coding sequence of PAS domain of KoBdcB, Km^r^ | This study |
| pET-28a (+)-KoBpdA^PAS^ | pET-28a (+) harboring the coding sequence of PAS domain of KoBpdA, Km^r^ | This study |
| pKT25 | Bacterial two-hybrid plasmid; p15A ori, Kan^r^, P_lac_, T25 | [3] |
| pUT18C | Bacterial two-hybrid plasmid; ColE1 ori, Amp^r^, P_lac_, T18 | [3] |
| pKT25-zip | Leucine zipper of GCN1 cloned into pKT25 for bacterial two-hybrid positive control; Kan^r^ | [3] |
| pUT18C-zip | Leucine zipper of GCN1 cloned into pUT18C for bacterial two-hybrid positive control; Amp^r^ | [3] |
| pKT25-KoBdcA | pKT25 with the coding sequence of KoBdcA; Km^r^ | This study |
| pUT18C-KoBdcA | pUT18C with the coding sequence of KoBdcA; Amp^r^ | This study |
| pKT25-KoBdcB | pKT25 with the coding sequence of KoBdcB; Km^r^ | This study |
| pUT18C-KoBdcB | pUT18C with the coding sequence of KoBdcB; Amp^r^ | This study |
| pKT25-KoBpdA | pKT25 with the coding sequence of KoBpdA; Km^r^ | This study |
| pUT18C-KoBpdA | pUT18C with the coding sequence of KoBpdA; Amp^r^ | This study |
| pKT25-BcsA | pKT25 with the coding sequence of the BcsA subunit of cellulose synthase | This study |
| pUT18C-BcsA | pUT18C with the coding sequence of the BcsA subunit of cellulose synthase | This study |
| pKT25- KoBdcA^PAS^ | pKT25 with the coding sequence of the PAS domain of KoBdcA | This study |
| pUT18C- KoBdcA^PAS^ | pUT18C with the coding sequence of the PAS domain of KoBdcA | This study |
| pKT25-KoBdcB^PAS^ | pKT25 with the coding sequence of the PAS domain of KoBdcB | This study |
| pUT18C-KoBdcB^PAS^ | pUT18C with the coding sequence of the PAS domain of KoBdcB | This study |
| pKT25-KoBpdA^PAS^ | pKT25 with the coding sequence of the PAS domain of KoBpdA | This study |
| pUT18C-KoBpdA^PAS^ | pUT18C with the coding sequence of the PAS domain of KoBpdA | This study |
| pKT25- KoBdcA^∆PAS^ | pKT25 with the coding sequence excluding the PAS domain of KoBdcA | This study |
| pUT18C- KoBdcA^∆PAS^ | pUT18C with the coding sequence excluding the PAS domain of KoBdcA | This study |
| pKT25-KoBdcB^∆PAS^ | pKT25 with the coding sequence excluding the PAS domain of KoBdcB | This study |
| pUT18C-KoBdcB^∆PAS^ | pUT18C with the coding sequence excluding the PAS domain of KoBdcB | This study |
| pKT25-KoBpdA^∆PAS^ | pKT25 with the coding sequence excluding the PAS domain of KoBpdA | This study |
| pUT18C-KoBpdA^∆PAS^ | pUT18C with the coding sequence excluding the PAS domain of KoBpdA | This study |

Table S2. The primers used in this study^a^

| Primers | Sequence (5' to 3') | Function |
| --- | --- | --- |
| KoBdcA-F1 | GGATAACTGGCAACGCCAATACGCTGGCGTAAATAATTCGCCC | gene knock-out |
| KoBdcA-R1 | AATTTTTTTAAGGCAGTTATTGGTGCGTGGATGCAGTTTACAAAGCC | gene knock-out |
| KoBdcA-F2 | GGAAGCATAAAGTCTCGAGATAAATCACGGCCAAATTTCTTTGGAAAGT | gene knock-out |
| KoBdcA-R2 | GGGCGAATTATTTACGCCAGCGTATTGGCGTTGCCAGTTATCC | gene knock-out |
| KoBdcB-F1 | GGAAGCATAAAGTCTCGAGATAAATCACGTTAATGATAGCCCACGCC | gene knock-out |
| KoBdcB-R1 | GCATAGAGCGCGGTATCAGCGTCTTCATCCCTGTGGTCTGTC | gene knock-out |
| KoBdcB-F2 | GACAGACCACAGGGATGAAGACGCTGATACCGCGCTCTATGC | gene knock-out |
| KoBdcB-R2 | AATTTTTTTAAGGCAGTTATTGGTGGCACTCCGCCACGAAATACC | gene knock-out |
| KoBpdA-F1 | GGAAGCATAAAGTCTCGAGATAAATCGTTTTTCGTCGCTGGCGTCGGAG | gene knock-out |
| KoBpdA-R1 | GCCACTGCTACCGTGCAAACTCACTCATCGATAGCGCCAAACAACGGAATGGT | gene knock-out |
| KoBpdA-F2 | ACCATTCCGTTGTTTGGCGCTATCGATGAGTGAGTTTGCACGGTAGCAGTGGC | gene knock-out |
| KoBpdA-R2 | AATTTTTTTAAGGCAGTTATTGGTGCTACTTACCAGGATCTGCTCTGGCCC | gene knock-out |
| KoBdcA-CF | CCATCACGAGATTTCGATTCCACCGTGCTGGAGCTGTTTAACTGGG | gene complementation |
| KoBdcA-CR | CTCATCGCAGTCGGCCTATTTCAACACACAACGGTGTTAATAATGCT | gene complementation |
| KoBdcB-CF | CCATCACGAGATTTCGATTCCACCGGGATAACGAAAAAGCCTGATGC | gene complementation |
| KoBdcB-CR | CTCATCGCAGTCGGCCTATTTCAGGGAACGGTGTAGGCAT | gene complementation |
| KoBpdA-CF | CCATCACGAGATTTCGATTCCACCCGGGGTGTGAGAGGTAAAATTTATC | gene complementation |
| KoBpdA-CR | CTCATCGCAGTCGGCCTATTTTAAATCATTTCACTTTGCGGCCTG | gene complementation |
| KoBdcA-PAS-F1 | GTGGACAGCAAATGGGTCGCATGTTTAATGAGATCGTGGTTTCAG | protein purification |
| KoBdcA-PAS-R1 | CAGTGGTGGTGGTGGTGGTGCAATTTTTCACGAATCGTCATATGTC | protein purification |
| KoBdcB-PAS-F1 | GTGGACAGCAAATGGGTCGCATGAGTGAAAAAAGACGGACAGACCACAGG | protein purification |
| KoBdcB-PAS-R1 | CAGTGGTGGTGGTGGTGGTGACTCGCCAGTTTCTGCTCGGCG | protein purification |
| KoBpdA-PAS-F1 | GTGGACAGCAAATGGGTCGCATGCATTCTGAAAATGATATTTTCGGCTCT | protein purification |
| KoBpdA-PAS-R1 | CAGTGGTGGTGGTGGTGGTGATGCTCGCTACTGGTCATCATCTGA | protein purification |
| pKoBdcA-F1 | ACAAAAGCTGGGTACCGGGCATGTTTAATGAGATCGTGGTTTCAGG | enzyme assay |
| pKoBdcA-R1 | GAATTGGAGCTCCACCGCGGACACACAACGGTGTTAATAATGCT | enzyme assay |
| pKoBdcB-F1 | ACAAAAGCTGGGTACCGGGCATGCATTCTGAAAATGATATTTTCGGCTCT | enzyme assay |
| pKoBdcB-R1 | GAATTGGAGCTCCACCGCGGATCATTTCACTTTGCGGCCTGC | enzyme assay |
| pKoBpdA-F1 | ACAAAAGCTGGGTACCGGGCATGAGTGAAAAAAGACGGACAGACCA | enzyme assay |
| pKoBpdA-R1 | GAATTGGAGCTCCACCGCGGATCATTTCACTTTGCGGCCTGC | enzyme assay |
| pKoBdcA- GGDEF-F | ACAAAAGCTGGGTACCGGGCCGTGCGCATAACGATGCATTAAC | enzyme assay |
| pKoBdcA-GGDEF-R | GAATTGGAGCTCCACCGCGGACACACAACGGTGTTAATAATGCT | enzyme assay |
| pKoBdcB- GGDEF-F | ACAAAAGCTGGGTACCGGGCGCCAACAAAGTGCTGCAACAATATGC | enzyme assay |
| pKoBdcB-GGDEF-R | GAATTGGAGCTCCACCGCGGGGGAACGGTGTAGGCATCCCG | enzyme assay |
| pKoBdcA-AAL-F | CTTGATAGTACGGCGACCGTGTTTTTTTTAATCGCG | enzyme assay |
| pKoBdcA-AAL-R | CGCGATTAAAAAAAACACGGCGCCGTACTATCAAG | enzyme assay |
| pKoBdcA-F2 | AATCGAAACAGTTAACGCCATGGAGGACAAT | construction of mutants |
| pKoBdcA-R2 | ATTGTCCTCCATGCCGTTAACTGTTTCGATT | construction of mutants |
| pKoBpdA-F2 | CGAAAATGTTGCCGATGCTGTCGTAC | construction of mutants |
| pKoBpdA-R2 | GTACGACAGCATCGGCAACATTTTCG | construction of mutants |
| KoBdcA-25-F | TGCAGGGTCGACTCTAGAGGATATGTTTAATGAGATCGTGGTTTCAGG | bacterial two-hybrid |
| KoBdcA-25-R | AAAACGACGGCCGAATTCTTAGTTAACACACAACGGTGTTAATAATGCT | bacterial two-hybrid |
| KoBdcA-18-F | TCTAGAGGATCCCCGGGTACCGATGTTTAATGAGATCGTGGTTTC | bacterial two-hybrid |
| KoBdcA-18-R | ACTGAGAGTGCACCATATTACTTAGTTATCAACACACAACGGTGTTAA | bacterial two-hybrid |
| KoBdcA-PAS-25-F | TGCAGGGTCGACTCTAGAGGATATGTTTAATGAGATCGTGGTTTCAG | bacterial two-hybrid |
| KoBdcA-PAS-25-R | AAAACGACGGCCGAATTCTTAGTTACAATTTTTCACGAATCGTCATATGT | bacterial two-hybrid |
| KoBdcA-PAS-18-F | TCTAGAGGATCCCCGGGTACCGATGTTTAATGAGATCGTGGTTTCAG | bacterial two-hybrid |
| KoBdcA-PAS-18-R | ACTGAGAGTGCACCATATTACTTAGTTACAATTTTTCACGAATCGTCATATGT | bacterial two-hybrid |
| KoBdcA-GD-25-F | TGCAGGGTCGACTCTAGAGGATATTCGTGAAAAATTGCATTTCC | bacterial two-hybrid |
| KoBdcA-GD-25-R | AAAAACGACGGCCGAATTCTTAGTTATCAACACACAACGGTGTTAA | bacterial two-hybrid |
| KoBdcA-GD-18-F | TCTAGAGGATCCCCGGGTACCGATTCGTGAAAAATTGCATTTCC | bacterial two-hybrid |
| KoBdcA-GD-18-R | ACTGAGAGTGCACCATATTACTTAGTTATCAACACACAACGGTGTTAA | bacterial two-hybrid |
| KoBdcB-25-F | TGCAGGGTCGACTCTAGAGGATATGAGTGAAAAAAGACGGACAGAC | bacterial two-hybrid |
| KoBdcB-25-R | AAAACGACGGCCGAATTCTTAGTTATCAGGGAACGGTGTAGGCAT | bacterial two-hybrid |
| KoBdcB-18-F | TCTAGAGGATCCCCGGGTACCGATGAGTGAAAAAAGACGGACAGACC | bacterial two-hybrid |
| KoBdcB-18-R | ACTGAGAGTGCACCATATTACTTAGTTATCAGGGAACGGTGTAGGCATC | bacterial two-hybrid |
| KoBdcB-PAS-25-F | TGCAGGGTCGACTCTAGAGGATATGAGTGAAAAAAGACGGACA | bacterial two-hybrid |
| KoBdcB-PAS-25-R | AAAACGACGGCCGAATTCTTAGTTACATCCATATAGCGGCGATTAA | bacterial two-hybrid |
| KoBdcB-PAS-18-F | TCTAGAGGATCCCCGGGTACCGATGAGTGAAAAAAGACGGACA | bacterial two-hybrid |
| KoBdcB-PAS-18-R | TTAATCGCCGCTATATGGATGTAACTAAGTAATATGGTGCACTCTCAGT | bacterial two-hybrid |
| KoBdcB-GD-25-F | TGCAGGGTCGACTCTAGAGGATCGGAGATCACCCGCGCCAT | bacterial two-hybrid |
| KoBdcB-GD-25-R | AAAAACGACGGCCGAATTCTTAGTTATCAGGGAACGGTGTAGGCATCCC | bacterial two-hybrid |
| KoBdcB-GD-18-F | TCTAGAGGATCCCCGGGTACCGCGGAGATCACCCGCGCCAT | bacterial two-hybrid |
| KoBdcB-GD-18-R | ACTGAGAGTGCACCATATTACTTAGTTATCAGGGAACGGTGTAGGCATCCCG | bacterial two-hybrid |
| KoBpdA-25-F | TGCAGGGTCGACTCTAGAGGATGTGCTGTCCTGCGAAGCA | bacterial two-hybrid |
| KoBpdA-25-R | TAAAACGACGGCCGAATTCTTAGTTATTAAATCATTTCACTTTGCGGCCTG | bacterial two-hybrid |
| KoBpdA-18-F | TCTAGAGGATCCCCGGGTACCGGTGCTGTCCTGCGAAGCA | bacterial two-hybrid |
| KoBpdA-18-R | ACTGAGAGTGCACCATATTACTTAGTTATTAAATCATTTCACTTTGCGGCCTG | bacterial two-hybrid |
| KoBpdA-PAS-25-F | TGCAGGGTCGACTCTAGAGGATATGCATTCTGAAAATGATATTTTCGGCTCT | bacterial two-hybrid |
| KoBpdA-PAS-25-R | AAAAACGACGGCCGAATTCTTAGTTAATGCTCGCTACTGGTCATCATCTGA | bacterial two-hybrid |
| KoBpdA-PAS-18-F | TCTAGAGGATCCCCGGGTACCGGTGCTGTCCTGCGAAGCA | bacterial two-hybrid |
| KoBpdA-PAS-18-R | ACTGAGAGTGCACCATATTACTTAGTTAATGCTCGCTACTGGTCATCATCTGA | bacterial two-hybrid |
| KoBpdA-GD-25-F | TGCAGGGTCGACTCTAGAGGATGCCCGCGCGGCGTTCTTCTTTCT | bacterial two-hybrid |
| KoBpdA-GD-25-R | AAAAACGACGGCCGAATTCTTAGTTAAGTGCTGAAGAACAGGTAACCGCCGGA | bacterial two-hybrid |
| KoBpdA-GD-18-F | TCTAGAGGATCCCCGGGTACCGGCCCGCGCGGCGTTCTTCTTTCT | bacterial two-hybrid |
| KoBpdA-GD-18-R | ACTGAGAGTGCACCATATTACTTAGTTAAGTGCTGAAGAACAGGTAACCGCCGGA | bacterial two-hybrid |
| BcsA-25-F | TGCAGGGTCGACTCTAGAGGATGTGCAAGATGATACAAACATGAAAA | bacterial two-hybrid |
| BcsA-25-R | AAAACGACGGCCGAATTCTTAGTTAAAGCGCATTGTTAACCTC | bacterial two-hybrid |
| BcsA-18-F | TCTAGAGGATCCCCGGGTACCGGTGCAAGATGATACAAACATGAAAAAAA | bacterial two-hybrid |
| BcsA-18-R | ACTGAGAGTGCACCATATTACTTAGTTAAAGCGCATTGTTAACCTCTTTCTG | bacterial two-hybrid |
|  |  |  |

^a^ Single and double underlined region in the primers represent overlap sequences and mutant sites, respectively.

References

[1] L. Hao, X. Liu, H. Wang, J. Lin, X. Pang, J. Lin, Detection and validation of a small broad-host-range plasmid pBBR1MCS-2 for use in genetic manipulation of the extremely acidophilic Acidithiobacillus sp, Journal of Microbiological Methods 90(3) (2012) 309-314.

[2] M. Wu, J. Qu, X. Tian, X. Zhao, Y. Shen, Z. Shi, P. Chen, G. Li, T. Ma, Tailor-made polysaccharides containing uniformly distributed repeating units based on the xanthan gum skeleton, International Journal of Biological Macromolecules 131 (2019) 646-653.

[3] G. Karimova, J. Pidoux, A. Ullmann, D. Ladant, A bacterial two-hybrid system based on a reconstituted signal transduction pathway, Proceedings of the National Academy of Sciences 95(10) (1998) 5752-5756.
